# Healthcare Deserts and Avoidable Mortality in Mexico: A Municipal-Level Ecological Analysis of Health System Resources, Social Deprivation, and Preventable Deaths, 2015–2024

**DOI:** 10.3390/healthcare14070890

**Published:** 2026-03-31

**Authors:** Ana María López-Yáñez, Judith Carolina De Arcos-Jiménez, Luis Fernando Herrera-Fuentes, Mauricio Alfredo Ambriz-Alarcón, Brian Rafael Rubio-Mora, Sofía Gutierrez-Perez, Violeta Cassandra Vera-Cuevas, Martha Cecilia Ledezma-Ramirez, Jaime Briseno-Ramirez

**Affiliations:** 1 Hospital Civil de Oriente, Tónala 45425, Mexico; ana.lopez@academicos.udg.mx (A.M.L.-Y.); judith.dearcos@academicos.udg.mx (J.C.D.A.-J.); lfherrera@hcg.gob.mx (L.F.H.-F.); martha.ledezma@academicos.udg.mx (M.C.L.-R.); 2 Departamento de Clinicas, Centro Universitario de Tlajomulco, Universidad de Guadalajara, Tlajomulco de Zuñiga 45641, Mexico; 3 Antiguo Hospital Civil de Guadalajara “Fray Antonio Alcalde”, Guadalajara 44280, Mexico; mambriz@hcg.gob.mx (M.A.A.-A.); brubio@hcg.gob.mx (B.R.R.-M.); 4 Comisión Estatal de Derechos Humanos Jalisco, Guadalajara 44600, Mexico; sofiagutierrezperez1982@gmail.com; 5 Centro de Investigaciones Biológicas del Noroeste, S. C., La Paz 23096, Mexico; vvera@pg.cibnor.mx

**Keywords:** healthcare deserts, avoidable mortality, preventable deaths, hospital infrastructure, social deprivation, spatial clustering, ecological study, Mexico, health inequalities, COVID-19

## Abstract

**Highlights:**

**What are the main findings?**

- Municipalities lacking hospital beds (63% of Mexico) had 43% higher avoidable mortality, independent of social deprivation.
- The COVID-19 pandemic widened the desert–adequate mortality *gap* by five fold (from ∼12 to 69 per 100,000), and rates had not recovered by 2024.

**What is the implication of the main finding?**

- Targeted hospital infrastructure expansion in southern Mexican healthcare deserts could substantially reduce avoidable deaths.
- Resource allocation policies should integrate healthcare desert status alongside social deprivation indices for equitable health planning.

**Abstract:**

**Background/Objectives:** Avoidable mortality—deaths before age 75 from preventable or treatable causes—is a key indicator of health system performance. In Mexico, nearly two-thirds of municipalities lack hospital beds, yet no study has examined the municipal-level association between healthcare infrastructure and avoidable mortality. This study assessed whether healthcare desert status is independently associated with avoidable mortality after adjusting for social deprivation. **Methods:** This ecological study analyzed 1891 Mexican municipalities (population ≥ 1000) over 2015–2024. Avoidable deaths were classified per OECD/Eurostat criteria (January 2022 revision). Healthcare desert status was defined by municipal hospital bed availability from 2019 facility data. Negative binomial mixed-effects regression estimated incidence-rate ratios (IRRs) adjusted for social deprivation, age structure, and state-level heterogeneity. Interrupted time-series analysis quantified pandemic disruption. **Results:** Of 4,960,244 deaths under 75 years, 81.2% were avoidable. Of 1891 municipalities, 1187 (62.8%) lacked hospital beds (healthcare deserts). Desert municipalities had 42.5% higher avoidable mortality (IRR = 1.425; 95% CI: 1.370–1.482; pre-pandemic 2015–2019), which attenuated to 1.353 after age-structure adjustment. Each standard-deviation increase in hospital beds (1 SD ≈ 2.2 beds per 1000) was associated with 7.9% lower mortality (IRR = 0.921). Avoidable mortality exhibited strong spatial clustering (Moran’s I = 0.382) in southern Mexico. By 2024, the desert–adequate mortality gap had widened by approximately five fold (from ∼12 to 69 per 100,000 population). **Conclusions:** Healthcare deserts are independently associated with substantially higher avoidable mortality in Mexico. The COVID-19 pandemic durably amplified pre-existing disparities associated with healthcare infrastructure deficits. Targeted hospital expansion in underserved municipalities is urgently needed, alongside investment in social determinants of health.

## 1. Introduction

Avoidable mortality—defined as deaths before age 75 from causes amenable to either public health interventions (preventable) or timely and effective healthcare (treatable)—has been widely adopted as an indicator of health system performance [1,2]. The concept, originally proposed by Rutstein et al. in 1976 as “sentinel health events” [3], was subsequently refined by Nolte and McKee, who demonstrated that cross-national differences in amenable mortality more accurately reflect healthcare system effectiveness than aggregate life expectancy [4]. The joint OECD/Eurostat classification, most recently revised in January 2022, provides a standardized framework encompassing over 170 ICD-10 codes for international comparisons [1]. In the European Union alone, an estimated 1.1 million avoidable deaths occurred in 2022, with substantial variation across countries [5]. Globally, a recent analysis comparing avoidable mortality across states of the United States and 40 high-income countries found widening disparities, underscoring the role of systemic health system factors in shaping population-level mortality patterns [6]. In low- and middle-income countries (LMICs), avoidable mortality accounts for an even larger share of premature deaths. Kruk et al. estimated that 8.6 million excess deaths annually were amenable to healthcare in 137 countries, of which 5.0 million were attributable to poor-quality care and 3.6 million to non-utilization of health services [7].

Geographic disparities in healthcare access represent a critical structural determinant of avoidable mortality. The concept of “healthcare deserts”—or “medical deserts” (*déserts médicaux*)—refers to geographic areas characterized by a severe shortage or complete absence of essential healthcare infrastructure, including hospital beds, physicians, and emergency services [8]. Although the term has been predominantly used in the context of high-income countries, with rural hospital closures in the United States and physician shortages in European regions having been linked to increased mortality [9,10], the concept is equally—if not more—relevant in LMICs, where health system fragmentation and resource maldistribution are endemic. At the population level, hospital bed density has been shown to be inversely associated with mortality; for instance, in China, the expansion of hospital bed supply was a significant determinant of maternal mortality reduction between 2004 and 2016 [11]. In Europe, ecological studies across 16 cities demonstrated significant associations between area-level deprivation and avoidable mortality, with the strongest gradients observed for treatable causes [12]. These findings support a resource–mortality nexus whereby inadequate healthcare infrastructure is associated with excess preventable and treatable deaths.

Mexico exemplifies the challenges faced by middle-income countries with fragmented health systems and profound geographic inequalities. The Mexican health system is segmented across multiple institutions—the Mexican Social Security Institute (IMSS), the Institute of Security and Social Services for State Workers (ISSSTE), and the Secretariat of Health (SSa)—each serving different population segments with unequal resource allocation [13,14]. Recent policy upheaval, including the dissolution of Seguro Popular and the subsequent transitions to INSABI and IMSS-Bienestar, has further disrupted coverage, with the share of uninsured individuals rising from 9% in 2018 to 26% in 2021 [15]. Mexico has the lowest hospital bed density in the OECD (1.0 per 1000 inhabitants), and this scarcity is unevenly distributed: approximately 60% of municipalities lack any hospital beds [16]. Previous studies have examined avoidable mortality in Mexico at the state level [17,18], and a recent systematic analysis found that over half of excess deaths in 2021 were amenable to healthcare, with 57% attributable to poor quality [19]. However, state-level analyses mask substantial within-state heterogeneity. In Latin America, city-level studies from the SALURBAL project have documented significant between-country variation in amenable mortality across 363 cities [20], yet sub-national ecological analyses at the municipal level—the smallest administrative division—remain scarce. Furthermore, no study has simultaneously assessed the independent associations of healthcare resource availability, social deprivation, and their spatial clustering patterns with avoidable mortality across all Mexican municipalities, nor examined how the COVID-19 pandemic differentially affected mortality trends by healthcare infrastructure status.

The objective of this study was to assess whether healthcare desert status is independently associated with age-standardized avoidable mortality rates across Mexican municipalities after adjusting for social deprivation. Secondary objectives included: (a) mapping of the geographic distribution of healthcare deserts and avoidable mortality, (b) quantification of the dose–response relationship between healthcare resource density and mortality, (c) identification of spatial clustering patterns through local indicators of spatial association, and (d) evaluation of temporal trends in avoidable mortality by healthcare infrastructure category across the period of 2015–2024, encompassing pre-pandemic, pandemic, and post-pandemic phases.

## 2. Materials and Methods

### 2.1. Study Design and Setting

This was an ecological study with a repeated cross-sectional design. The unit of analysis was the municipality (*municipio*), the smallest administrative division in Mexico. The study encompassed 2469 municipalities across 32 federal entities (states). The study period spanned 2015–2024 (10 years), segmented into three phases: pre-pandemic (2015–2019), pandemic (2020–2021), and post-pandemic (2022–2024). The primary analysis was based on pre-pandemic 5-year annual averages (2015–2019) to establish baseline associations free of COVID-19 confounding [21]. full-period analysis (2015–2024) was conducted as a sensitivity analysis to evaluate the robustness of associations when incorporating pandemic and post-pandemic years. Municipalities with populations below 1000 inhabitants were excluded from regression analyses to ensure stable rate estimation, yielding 1891 municipalities for the primary analysis.

### 2.2. Data Sources

#### 2.2.1. Mortality Data

Individual death records were obtained from the National Institute of Statistics and Geography (INEGI) through Mexico’s open data platform (https://www.datos.gob.mx/, accessed on 25 February 2025) for 2015–2023 and from the INEGI Registered Deaths Statistics program (EDR) for 2024 [22]. Each record includes the underlying cause of death coded according to the International Classification of Diseases, Tenth Revision (ICD-10); age; sex; and municipality of usual residence identified by a 5-digit geostatistical code. The dataset comprised 8,191,145 total death records across the 10-year period, of which 4,960,244 corresponded to deaths under 75 years of age.

#### 2.2.2. Healthcare Resources

Health facility-level data were obtained from the Sectorial Health Resources Database of the General Directorate of Health Information (DGIS) for the year 2019 [23]. This database contains all health-sector institutions—including the Mexican Social Security Institute (IMSS), the Institute for Social Security and Services for State Workers (ISSSTE), the Secretariat of Health (SSa), and private facilities—linked through the Unique Health Establishment Code (CLUES). Extracted variables included hospital beds, total physicians, nurses, outpatient clinics, and operating rooms. Data from 22,220 individual health facilities were aggregated to 2449 municipalities using the CLUES-to-municipality linkage. The 2019 cross-section was selected as the pre-pandemic infrastructure baseline.

#### 2.2.3. Social Deprivation

The Social Lag Index (*Índice de Rezago Social*, IRS) 2020 was obtained from the National Council for the Evaluation of Social Development Policy (CONEVAL) [24]. The IRS is a composite index derived from the first principal component of a set of socioeconomic indicators spanning six dimensions: educational attainment (illiteracy rate, incomplete primary education, and incomplete secondary education), health service access (population without health coverage), housing quality (dirt floors, inadequate walls, amd inadequate roofing), basic services (lack of piped water, lack of drainage, and lack of electricity), household assets (lack of washing machine and lack of refrigerator), and living space (overcrowding). The first principal component captures the dominant axis of correlated deprivation; CONEVAL standardizes the resulting scores to a mean of zero at the national level such that positive values indicate above-average social deprivation and negative values indicate below-average deprivation. Thus, higher values reflect greater cumulative disadvantage across multiple dimensions. The first principal component typically captures more than 50% of the total variance across the input indicators, confirming that these deprivation dimensions are highly correlated and can be meaningfully summarized by a single axis. CONEVAL publishes the IRS at national, state, municipal, and locality levels every five years, tied to census or intercensal survey data; the 2020 edition is based on the 2020 Population and Housing Census. Additionally, the municipal poverty rate (%) from CONEVAL’s 2020 multidimensional poverty measurement was included [24].

#### 2.2.4. Population Denominators

Municipal population projections by 5-year age group and sex were obtained from the National Population Council (CONAPO) [25]. These projections, available for 1990–2040, provided age-specific denominators for rate computation.

### 2.3. Variable Definitions

#### 2.3.1. Outcome: Avoidable Mortality Rate

Deaths were classified as avoidable according to the joint OECD/Eurostat list of preventable and treatable causes of death (January 2022 revision) [1]. This classification encompasses 170 ICD-10 code patterns, subdivided into preventable causes (amenable to public health interventions) and treatable causes (amenable to timely and effective healthcare). Deaths where a cause is partially attributable to both categories were assigned using the 50/50 split rule specified in the classification [1]. Only deaths occurring before age 75 were included, consistent with the OECD/Eurostat age threshold.

A two-level matching algorithm was applied: specific 4-character ICD-10 codes were matched first, followed by 3-character codes for unmatched records. Of the 4,960,244 deaths under age 75, 4,028,038 (81.2%) were classified as avoidable (50.2% preventable, 31.0% treatable).

Age-standardized mortality rates (ASR) were computed per 100,000 population using the direct method of standardization [26]. The WHO World Standard Population (Ahmad et al. 2001) was used as the reference, with weights normalized to the 0–74 age range across 15 five-year age groups [27]. Rates were computed as 5-year annual averages for the pre-pandemic period (2015–2019) and as 10-year annual averages for the full period (2015–2024). Sub-category rates were computed separately for preventable and treatable mortality.

#### 2.3.2. Exposure: Healthcare Desert Classification

A three-level healthcare desert classification was constructed based on municipal hospital bed availability from the 2019 DGIS resource database:

- Desert: No hospital beds in the municipality (0 beds);
- Limited: Hospital beds present but below the median bed density among municipalities with hospitals (median = 0.69 beds per 1000 inhabitants);
- Adequate (reference category): Hospital bed density at or above the median.

Additionally, continuous healthcare resource variables were included: hospital beds per 1000 population and physicians per 1000 population, both standardized as z-scores to enable comparison of effect sizes across variables.

#### 2.3.3. Covariates

Social deprivation was measured using the CONEVAL Social Lag Index 2020, standardized as a z-score (IRS-z). The continuous z-score specification was preferred over CONEVAL’s five-level categorical classification (Very Low through Very High) for regression modeling because it preserves the full distributional information, avoids arbitrary cutpoint selection, and enables direct comparison of effect sizes across standardized predictors [28]. To account for potential confounding by municipal age structure—given that the negative binomial count model with a population offset adjusts for population volume but not age composition—the proportion of the under-75 population aged 60–74 years was computed from CONAPO projections and included as a standardized covariate (prop-elderly-z). The 32 federal entities (states) were included as a grouping factor for multilevel modeling to account for state-level heterogeneity in health policies, governance, and unmeasured contextual factors.

### 2.4. Statistical Analysis

#### 2.4.1. Descriptive Analysis

Continuous variables were summarized as medians with interquartile ranges (IQRs), and categorical variables were summarized as frequencies with percentages. Kruskal–Wallis tests were used to compare ASR across the three healthcare desert categories, and Spearman rank correlations were computed between continuous predictors and ASR [29].

#### 2.4.2. Regression Modeling

The relationship between healthcare infrastructure, social deprivation, and avoidable mortality was modeled using negative binomial (NB) regression. The NB distribution was preferred over the Poisson distribution because the observed variance in avoidable death counts substantially exceeded the mean (Pearson dispersion ratio = 12.9), indicating overdispersion that would lead to underestimated standard errors and inflated Type I error rates under the Poisson assumption [28]. Under the NB parameterization, the variance of the outcome is given by

(1) $$\mathrm{Var}\left(Y_{i}\right)=\mu_{i}+\frac{\mu_{i}^{2}}{\theta }$$

where $\mu_{i}$ is the expected count and θ is the overdispersion parameter governing the degree of extra-Poisson variability; as θ→∞, the distribution converges to the Poisson.

For municipality *i*, the expected count of avoidable deaths was modeled as

(2) $$\log\left(\mu_{i}\right)=\log\left(N_{i}\right)+\beta_{0}+\sum_{k}\beta_{k}\;x_{ki}$$

where $N_{i}$ denotes the average annual population. The term $\log(N_{i})$ enters as an offset—a predictor with a coefficient fixed at 1—which effectively converts the modeled count into a rate by constraining the model to estimate $\log(\mu_{i}/N_{i})$ rather than $\log(\mu_{i})$. This ensures that regression coefficients are interpretable as log-rate ratios rather than log-count differences. $\beta_{0}$ is the intercept, and $x_{ki}$ represents the value of the *k*-th predictor for municipality *i*. Regression coefficients were exponentiated and expressed as incidence-rate ratios ($\mathrm{IRR}=e^{\beta }$), which quantify the multiplicative change in the avoidable mortality rate per unit change in each predictor. Wald-based 95% confidence intervals accompanied all estimates.

A sequential model-building strategy was employed to disentangle the relative contributions of social deprivation and healthcare resources. Model A included only the standardized Social Lag Index (IRS-z) as a single predictor, representing the social deprivation pathway. Model B included only healthcare resource variables—standardized physician density (docs-z), standardized hospital bed density (beds-z), and the three-level categorical desert classification—representing the healthcare supply pathway. Model C combined all predictors from Models A and B into a full fixed-effects specification to evaluate the independent contribution of each factor after mutual adjustment.

To account for unmeasured state-level heterogeneity in health policies, governance capacity, and epidemiological context, Model C2 extended Model C by incorporating a state-level random intercept. This generalized linear mixed model takes the following form:

(3) $$\log\left(\mu_{ij}\right)=\log\left(N_{ij}\right)+\beta_{0}+\sum_{k}\beta_{k}\;x_{kij}+u_{j},\;u_{j}∼N\left(0,\sigma_{u}^{2}\right)$$

where the subscript *j* indexes the 32 federal entities and $u_{j}$ captures the state-specific deviation from the overall intercept, assumed to follow a normal distribution with a variance of $\sigma_{u}^{2}$. The 32 federal entities were selected as the grouping level because they represent the primary administrative unit for health policy, governance, and resource allocation in Mexico. Health jurisdictions (*jurisdicciones sanitarias*) were considered but not used because jurisdiction boundaries are defined differently across states and may not correspond to meaningful epidemiological units, and jurisdiction-level covariate data were not consistently available. Model C2 was fitted by maximum likelihood using the Laplace approximation as implemented in the glmer.nb function [30] and was designated as the primary model. Finally, Model D augmented Model C by adding cross-product interaction terms between social deprivation (IRS-z) and the desert category to test whether the mortality effect of healthcare infrastructure deficit was modified by the level of social deprivation—that is, whether these determinants acted synergistically or additively.

An age structure-adjusted variant of Model C2 was additionally fitted, incorporating the standardized proportion of the population aged 60–74 years (prop-elderly-z) as a covariate. This specification accounts for the potential confounding introduced by differential age composition across municipalities, given that the population offset adjusts for volume but not age structure—a concern when descriptive statistics report age-standardized rates (ASRs) while the regression models count-based outcomes.

Model fit was compared using Akaike’s Information Criterion (AIC) and the Bayesian Information Criterion (BIC), with lower values indicating superior parsimony-adjusted fit. Multicollinearity among predictors was assessed via variance inflation factors (VIFs); values below 5 were considered acceptable [31].

#### 2.4.3. Spatial Analysis

To assess whether avoidable mortality exhibited non-random geographic patterns and to evaluate the robustness of regression estimates to spatial dependence, spatial autocorrelation analyses and spatial regression models were conducted at the municipal level. Municipal polygons (2469 municipalities) based on the INEGI geostatistical framework were obtained from the mxmaps R package. A queen contiguity spatial-weights matrix (W) was constructed, in which two municipalities are defined as neighbors if they share any boundary segment or vertex [32]. The elements of W were row-standardized such that the weights for each municipality’s neighbors sum to unity, yielding spatially lagged values interpretable as the weighted mean of neighboring observations. Island municipalities (those lacking contiguous neighbors) were handled using a zero-policy approach, effectively excluding them from the spatial lag computation.

Global spatial autocorrelation was evaluated using Moran’s I statistic [33], which ranges from approximately −1 (perfect dispersion) to +1 (perfect clustering). Statistical significance was assessed using 999 random permutations, computed separately for avoidable, preventable, and treatable ASR. Local indicators of spatial association (LISA) were computed using local Moran’s I [34] to identify spatial clusters (High–High, hot spots; Low–Low, cold spots) and outliers (High–Low, Low–High), retaining only clusters significant at p<0.05 after multiple-comparison adjustment.

To evaluate the robustness of regression estimates to residual spatial dependence, Moran’s I was computed on the Pearson residuals of the primary model (Model C2). Additionally, a spatial error model (SEM) was fitted using maximum likelihood estimation via the spatialreg R package [35], with log-transformed ASR as the outcome and the same predictor set as Model C. The SEM incorporates a spatially autocorrelated error term governed by a parameter (λ, the spatial autocorrelation coefficient), which captures the degree to which unobserved spatially structured factors influence the outcome. The statistical significance of λ was evaluated using a likelihood ratio test comparing the SEM to the corresponding OLS model. A spatial lag model (SAR) was also fitted as an alternative specification. Moran’s I was recomputed on the residuals of both spatial models to verify that the spatial structure had been adequately captured.

#### 2.4.4. Sensitivity Analyses

Six sensitivity analyses were conducted to evaluate the robustness of the primary findings. First, the regression analysis was repeated, restricting the outcome to treatable mortality only—that is, deaths from causes directly amenable to timely and effective healthcare—to isolate the association between healthcare infrastructure and the mortality component most sensitive to health service availability, excluding the preventable component that responds primarily to public health interventions rather than clinical care. Second, the full regression model specifications (Models C and C2) were replicated using 10-year period averages (2015–2024) rather than the pre-pandemic baseline alone to evaluate whether the observed associations persisted when incorporating the substantial mortality disruption caused by the COVID-19 pandemic and the subsequent post-pandemic recovery period. Third, yearly population-weighted mean ASR was computed by healthcare desert category across all 10 years (2015–2024), using the under-75 population as weights, to characterize the temporal dynamics of avoidable mortality by infrastructure status—including the pre-pandemic baseline, the pandemic surge, the differential impact across healthcare categories, and the trajectory of post-pandemic recovery. Fourth, to address the potential temporal mismatch between the 2019 healthcare resource cross-section and the 2015–2019 mortality period, Model C2 was re-estimated, restricting the outcome to the 2018–2019 biennium average, when resource data and mortality data are temporally aligned. Fifth, the spatial error model described in Section 2.4.3 was conducted to assess whether the primary associations were robust to explicit modeling of spatial autocorrelation. Sixth, to empirically investigate the counterintuitive positive association between physician density and mortality (the “physician paradox”), an exploratory model was fitted, incorporating log-transformed municipal population as a proxy for urbanicity, alongside the proportion of the elderly population, to test whether the physician effect was confounded by the urban concentrations of both physicians and referral deaths.

#### 2.4.5. Interrupted Time-Series Analysis

To formally quantify the pandemic disruption and subsequent recovery, an interrupted time-series (ITS) analysis was conducted using segmented negative binomial regression at the municipality level. Monthly avoidable death counts were computed for each of the 1891 municipalities over the period of January 2015 to December 2024; after excluding municipality months with zero estimated population denominators, the final unbalanced panel comprised 216,512 municipality-month observations. The intervention was defined as March 2020, corresponding to the World Health Organization’s declaration of the COVID-19 pandemic and Mexico’s first documented cases. The segmented regression model took the following form:

(4) $$\begin{array}{c} \log\left(\mu_{it}\right)=\log\left(N_{it}\right)+\beta_{0}+\beta_{1}\;\cdot \;{\mathrm{time}}_{t}+\beta_{2}\;\cdot \;{\mathrm{intervention}}_{t}+\beta_{3}\;\cdot \;{\mathrm{time}\text{\_}\mathrm{since}}_{t}\\ +\gamma_{1}\sin\;\left(\frac{2\pi t}{12}\right)+\gamma_{2}\cos\;\left(\frac{2\pi t}{12}\right)+\gamma_{3}\sin\;\left(\frac{2\pi t}{6}\right)+\gamma_{4}\cos\;\left(\frac{2\pi t}{6}\right) \end{array}$$

where $N_{it}$ is the monthly population offset, ${\mathrm{time}}_{t}$ is a continuous index (months 1–120), ${\mathrm{intervention}}_{t}$ is a binary indicator for March 2020 onward, and ${\mathrm{time}\text{\_}\mathrm{since}}_{t}$ counts months elapsed since the intervention. Fourier harmonic pairs at 12-month and 6-month periodicities ($\gamma_{1}$–$\gamma_{4}$) controlled for seasonal variation. In this specification, $\beta_{1}$ captures the pre-pandemic trend (monthly rate of change), $\beta_{2}$ estimates the immediate level change at the pandemic onset, and $\beta_{3}$ estimates the post-intervention slope change. On the IRR scale, the total post-pandemic monthly trend equals $\exp\left(\beta_{1}+\beta_{3}\right)={\mathrm{IRR}}_{\beta_{1}}\times {\mathrm{IRR}}_{\beta_{3}}$, since on the log scale, the post-pandemic slope is $\beta_{1}+\beta_{3}$. Standard errors were computed using cluster-robust sandwich estimators at the municipality level [36]. Separate stratified models were fitted for each desert category, and a pooled interaction model with cross-product terms between desert category and both $\beta_{2}$ and $\beta_{3}$ formally tested whether pandemic impact and recovery differed across categories (Supplementary Table S8). Counterfactual rates were estimated by setting the intervention and post-intervention terms to zero.

### 2.5. Software

All analyses were performed in R version 4.4.1 [37]. Key packages (with versions) included data.table 1.18.2 for large-scale data management [38]; MASS 7.3-65 (glm.nb) and lme4 1.1-38 (glmer.nb) for negative binomial regression [30,39]; spdep 1.4-2 and spatialreg 1.4-2 for spatial weights, Moran’s I, LISA, and spatial error/lag models [35,40]; sandwich 3.1-1 for cluster-robust standard errors [36]; sf 1.1-0 for geospatial operations [41]; and ggplot2 4.0.2, patchwork 1.3.2, and mxmaps 2020.2.2 for visualization [42,43,44]. Analysis scripts are available at https://doi.org/10.5281/zenodo.19241171.

### 2.6. Ethical Statement

This study used exclusively publicly available, de-identified, aggregated data from official open government sources (INEGI, DGIS, CONEVAL, and CONAPO). No individual-level identifiable information was accessed or used. In accordance with Mexican regulations and institutional guidelines, ethics committee approval was not required for ecological analyses based on open administrative data.

## 3. Results

### 3.1. Study Population

**Figure 1:**
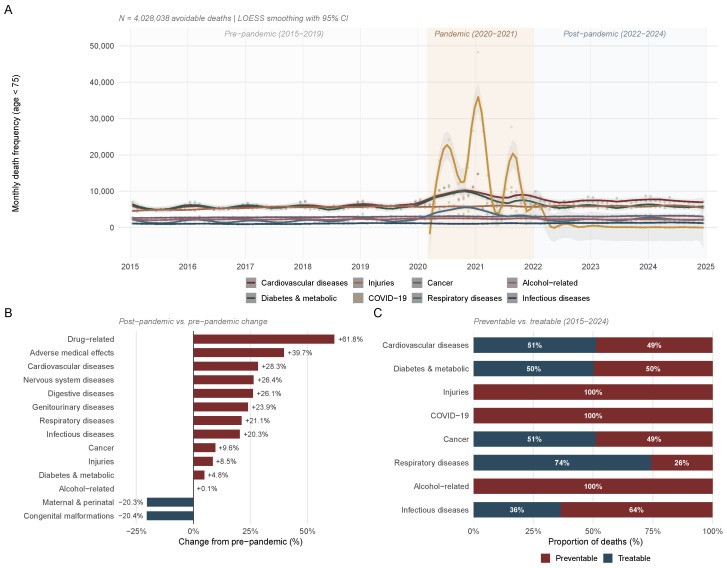
Cause-specific avoidable mortality trends among adults under 75 years, Mexico 2015–2024. Panel (**A**): Monthly death frequencies for the top eight cause groups with locally estimated scatterplot smoothing (LOESS, span = 0.15) and 95% confidence intervals, showing the COVID-19 surge in 2020–2021 and persistent elevations in cardiovascular and diabetes-related mortality post-pandemic. Panels (**B**,**C**): Preventable and treatable mortality components by cause group across three phases (pre-pandemic, 2015–2019; pandemic, 2020–2021; post-pandemic, 2022–2024). Underlying weekly frequencies by cause group are provided in Supplementary Figure S1. Avoidable mortality classified per the OECD/Eurostat 2022 framework [1]. Source: INEGI mortality microdata [22]; population denominators from CONAPO projections [25].

Of 2469 Mexican municipalities, 1891 met the inclusion criterion (population ≥ 1000) and were retained for the primary pre-pandemic analysis. The mean annual municipal population was 57,368 (SD = 159,409), with a median of 12,775 (IQR: 4617–37,947). Across the 10-year study period (2015–2024), 4,960,244 deaths occurred among individuals younger than 75 years, of which 4,028,038 (81.2%) were classified as avoidable according to the OECD/Eurostat criteria [1]. The pre-pandemic (2015–2019) overall age-standardized avoidable mortality rate (ASR) was 767.2 per 100,000 population (SD = 162.9), comprising preventable mortality (466.4 per 100,000; SD = 112.1) and treatable mortality (300.8 per 100,000; SD = 68.5). Figure 1 presents cause-specific temporal dynamics: cardiovascular diseases accounted for the largest share of avoidable deaths (20.7% pre-pandemic), followed by diabetes and metabolic disorders (20.3%) and injuries (19.5%). The pandemic dramatically altered the cause distribution, with COVID-19 representing 27.6% of avoidable deaths during 2020–2021 (Supplementary Table S1). Post-pandemic, annual averages for most cause groups exceeded pre-pandemic levels, with respiratory diseases (+21.1%), genitourinary diseases (+23.9%), and cardiovascular diseases (+28.3%) showing the largest persistent increases (Figure 1B,C).

### 3.2. Healthcare Resource Distribution

**Figure 2:**
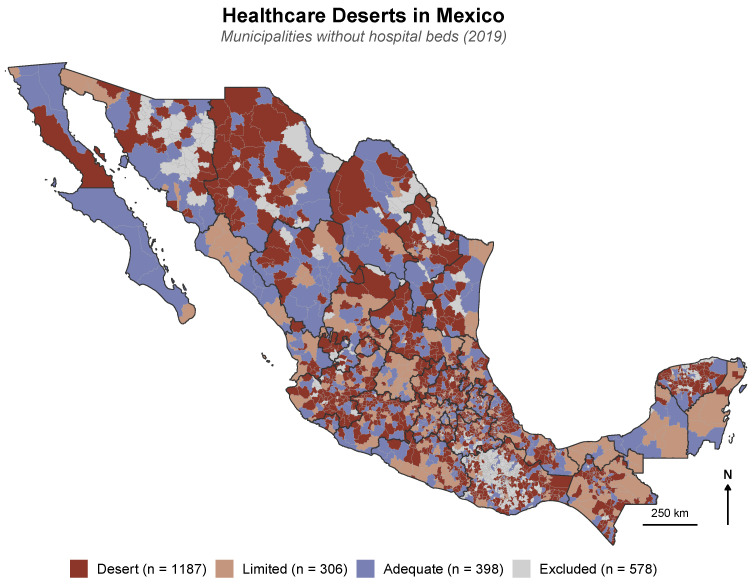
Geographic distribution of healthcare desert classification across 1891 Mexican municipalities. Desert = no hospital beds (n=1187; 62.8%); Limited = hospital beds below median of 0.69 per 1000 population (n=306; 16.2%); Adequate = hospital beds at or above median (n=398; 21.0%). Classification based on 2019 DGIS Sectorial Resources data [23].

Of the 1891 municipalities, 1187 (62.8%) were classified as healthcare deserts, 306 (16.2%) as limited, and 398 (21.0%) as adequate (Figure 2). Table 1 presents the descriptive characteristics of municipalities by healthcare desert category (Supplementary Table S2 provides the complete variable set). Healthcare deserts had no hospitals (median beds per 1000 = 0.0), compared with a median of 1.2 per 1000 in adequate municipalities. Physician density followed a similar gradient: a median pf 1.0 per 1000 in deserts versus 2.3 per 1000 in adequate municipalities. Desert municipalities exhibited greater social deprivation (mean Social Lag Index of 0.06 vs. −0.43) and higher poverty rates (median of 63.2% vs. 50.0%). The median population in deserts (6881) was markedly smaller than in adequate municipalities (29,453), reflecting the predominantly rural character of healthcare deserts. All differences across categories were statistically significant (p<0.001; Table 1).

### 3.3. Bivariate Associations

Table 2 summarizes the bivariate associations between predictors and age-standardized mortality rates. Kruskal–Wallis tests revealed that bivariate differences in ASR across healthcare desert categories were modest and not statistically significant for avoidable (p=0.075) or preventable mortality (p=0.155), though treatable mortality retained significance ($\chi^{2}=8.10$, df = 2, p=0.017; Table 2, Panel A). This non-significance at the bivariate level reflects the well-known limitation of unadjusted ecological comparisons: healthcare deserts tend to be small, rural municipalities with younger age structures and lower absolute death counts, and confounders such as physician density and population size mask the underlying infrastructure–mortality relationship. As shown in Section 3.4, multivariable regression adjusting for these confounders reveals a highly significant association (desert IRR = 1.425; p<0.001).

Spearman correlations (Table 2, Panel B) revealed an inverse association between physician density and both ASR avoidable (ρ=−0.179, p<0.001) and ASR treatable (ρ=−0.201, p<0.001). Poverty rate was positively correlated with avoidable mortality (ρ=0.111, p<0.001) but not with treatable mortality (ρ=0.009, p=0.681). Hospital bed density showed a negligible bivariate correlation with both outcomes (ρ=0.003 and 0.004, respectively), a finding that was reversed in multivariable models after adjustment for confounding—most notably, population size and physician density, which are strongly correlated with bed availability (Supplementary Table S3) and create opposing forces that mask the protective effect at the bivariate level. Notably, the Social Lag Index exhibited opposing associations: positive with avoidable mortality (ρ=0.065, p=0.005) but negative with treatable mortality (ρ=−0.127, p<0.001), suggesting differential confounding patterns across mortality subcategories. The full Spearman correlation matrix among all continuous variables is provided in Supplementary Table S3.

**Table 2:** Bivariate associations between healthcare resources, social deprivation, and age-standardized mortality rates across 1891 municipalities (pre-pandemic 2015–2019).

**Panel A. Kruskal–Wallis tests by healthcare desert category**						
**Outcome**	***χ* ^2^**		**df**		***p*-Value**	
ASR avoidable mortality	5.18		2		0.075	
ASR preventable mortality	3.73		2		0.155	
ASR treatable mortality	8.10		2		0.017	
**Panel B. Spearman rank correlations (ρ) with mortality rates**						
**Predictor**	**ASR Avoidable**	***p*-Value**	**ASR Preventable**	***p*-Value**	**ASR Treatable**	***p*-Value**
Hospital beds per 1000	0.003	0.893	0.003	0.882	0.004	0.849
Physicians per 1000	−0.179	<0.001	−0.133	<0.001	−0.201	<0.001
Social Lag Index	0.065	0.005	0.172	<0.001	−0.127	<0.001
Poverty rate (%)	0.111	<0.001	0.157	<0.001	0.009	0.681
Population	0.110	<0.001	0.037	0.112	0.204	<0.001

Panel A: Kruskal–Wallis tests comparing age-standardized rates across healthcare desert categories (Desert, Limited, and Adequate). The non-significant bivariate differences for avoidable and preventable mortality reflect confounding by municipal size, age structure, and physician density; multivariable regression (Table 3) reveals a highly significant association (desert IRR = 1.425; p<0.001). Panel B: Spearman rank correlation coefficients between continuous predictors and age-standardized mortality rates (per 100,000). ASR: age-standardized rate. Social Lag Index from CONEVAL 2020. The non-significant bivariate correlation for hospital beds reflects confounding by population size and referral-center effects (see Section 3.3); multivariable regression (Table 3) reveals a significant protective association after adjustment.

**Table 3:** Negative binomial regression models for age-standardized avoidable mortality: incidence-rate ratios (IRR) with 95% confidence intervals (pre-pandemic, 2015–2019; n=1891 municipalities).

Predictor	Model A	Model B	Model C	Model C2 ^†^	Model D
Social Lag Index (per SD)	1.080 (1.061–1.099) p<0.001	—	1.038 (1.022–1.053) p<0.001	1.025 (1.007–1.043) p=0.006	1.092 (1.056–1.129) p<0.001
Physicians per 1000 (per SD)	—	1.195 (1.162–1.228) p<0.001	1.196 (1.164–1.229) p<0.001	1.210 (1.178–1.243) p<0.001	1.197 (1.165–1.230) p<0.001
Hospital beds per 1000 (per SD)	—	0.948 (0.922–0.975) p<0.001	0.944 (0.918–0.971) p<0.001	0.921 (0.895–0.949) p<0.001	0.944 (0.918–0.971) p<0.001
Limited (vs. Adequate)	—	0.911 (0.870–0.954) p<0.001	0.907 (0.866–0.950) p<0.001	0.948 (0.907–0.992) p=0.021	0.883 (0.841–0.926) p<0.001
Desert (vs. Adequate)	—	1.477 (1.422–1.535) p<0.001	1.448 (1.393–1.506) p<0.001	1.425 (1.370–1.482) p<0.001	1.424 (1.368–1.481) p<0.001
Limited × IRS-z	—	—	—	—	0.913 (0.869–0.960) p<0.001
Desert × IRS-z	—	—	—	—	0.944 (0.909–0.981) p=0.003
AIC	17,781	17,093	17,075	16,817	17,067
BIC	17,798	17,127	17,114	16,862	17,116

Model specifications—A: social deprivation only; B: healthcare resources only; C: full model (fixed effects); C2: full model with state-level random intercept (primary model); D: full model with deprivation × desert category interaction. ^†^ Primary model selected based on lowest AIC. All models use log(average annual population) as offset. IRR = exp(β). Continuous predictors standardized as z-scores. Standardized predictor SD values: hospital beds per 1000 (SD = 2.2), physicians per 1000 (SD = 2.4), and Social Lag Index (SD = 1.00). Variance inflation factors < 4.1 for all predictors.

### 3.4. Regression Models—Primary Analysis (2015–2019)

Overdispersion in the Poisson model was confirmed (Pearson dispersion ratio = 12.9), justifying the use of negative binomial regression [28]. Table 3 presents the complete results of the sequential model-building strategy across five specifications. Model fit improved progressively from Model A (social deprivation only) through Model B (healthcare resources only) and from Model C (full fixed effects) to Model C2 (full model with state-level random intercept), which achieved the lowest AIC (16,817; Supplementary Table S4) and was selected as the primary model. The inclusion of state-level random intercepts substantially improved fit (ΔAIC = 258 between Models C and C2), indicating significant geographic heterogeneity in avoidable mortality beyond municipal-level predictors. The estimated state-level random intercept variance was ${\hat{\sigma }}_{u}^{2}=0.023$ (SD = 0.153), corresponding to an intraclass correlation coefficient (ICC) of approximately 0.007 under the latent-variable approximation (ICC = $\sigma_{u}^{2}/\left(\sigma_{u}^{2}+\pi^{2}/3\right)$), indicating that while the state-level variance is modest relative to total variation, it is nonetheless statistically meaningful and captures policy and governance differences across federal entities.

When entered as a single predictor (Model A), social deprivation was associated with 8.0% higher avoidable mortality per standard deviation increase (Table 3). When healthcare resource variables were modeled without adjustment for deprivation (Model B), desert status was associated with 47.7% higher mortality relative to adequate municipalities. After simultaneous adjustment for all covariates and state-level heterogeneity in the primary model (Model C2), healthcare desert status remained independently associated with 42.5% higher avoidable mortality (IRR = 1.425; 95% CI: 1.370–1.482; p<0.001). Each standard-deviation increase in hospital beds per capita (1 SD ≈ 2.2 beds per 1000) was associated with a 7.9% reduction in mortality (IRR = 0.921; p<0.001), while social deprivation retained an independent 2.5% increase per standard deviation (IRR = 1.025; p=0.006). Physician density exhibited a positive association with mortality (IRR = 1.210; p<0.001), a counterintuitive finding interpreted in the Discussion as confounding by indication.

Adjusting for municipal age structure (Model C2a, incorporating prop-elderly-z) modestly attenuated the desert association from IRR = 1.425 to 1.353 (95% CI: 1.312–1.396; p<0.001), while the proportion of the population aged 60–74 years emerged as a strong independent predictor (IRR = 1.220; 95% CI: 1.204–1.237; p<0.001; Supplementary Table S5). Model C2a improved fit relative to Model C2 (ΔAIC = −664), indicating that age composition explained meaningful variance in avoidable mortality beyond population volume. Hospital beds retained their protective effect (IRR = 0.932; p<0.001), and the physician paradox persisted (IRR = 1.146; p<0.001). The desert association remained robust after age-structure adjustment, confirming that the primary finding is not an artifact of differential age composition across municipalities.

Variance inflation factors for all predictors were below 4.1, confirming no problematic multicollinearity. In contrast to the additive pattern observed in the original submission, the interaction between social deprivation and desert status (Model D) was statistically significant for both desert × IRS-z (IRR = 0.944; 95% CI: 0.909–0.981; p=0.003) and limited × IRS-z (IRR = 0.913; 95% CI: 0.869–0.960; p<0.001). These sub-multiplicative interaction terms indicate that the marginal effect of social deprivation on avoidable mortality is attenuated in municipalities lacking hospital infrastructure. This pattern is consistent with a ceiling effect: in healthcare deserts, where the complete absence of hospital beds already dominates the mortality risk profile, additional social deprivation contributes less incrementally to excess mortality than in municipalities where hospital infrastructure is present and access barriers are primarily socioeconomic rather than structural.

### 3.5. Sensitivity Analysis: Treatable Mortality

Table 4 compares the primary pre-pandemic model with two sensitivity analyses. When the outcome was restricted to treatable causes—those directly amenable to timely and effective healthcare—the desert association remained robust (IRR = 1.342; 95% CI: 1.295–1.391; p<0.001). The protective effect of hospital beds was more pronounced for treatable mortality (IRR = 0.900; p<0.001) compared with all avoidable mortality (IRR = 0.921), consistent with the expectation that hospital infrastructure most directly impacts healthcare-amenable conditions. Social deprivation showed a non-significant association for treatable mortality (IRR = 1.001; p=0.942), and the physician density paradox persisted (IRR = 1.198; p<0.001; Table 4).

### 3.6. Sensitivity Analysis: Full Period of 2015–2024

When extending the analysis to all 10 years (2015–2024), all primary associations remained statistically significant (Table 4). The desert IRR was modestly attenuated from 1.425 to 1.404 (95% CI: 1.353–1.457; p<0.001), while the protective effect of hospital beds was similar (IRR = 0.933 vs. 0.921). Social deprivation was non-significant in the full-period model (IRR = 1.000; p=0.960), while the limited category was marginally non-significant (IRR = 0.959; p=0.054). The modest attenuation of the desert effect is consistent with the observation that COVID-19 mortality disproportionately affected urban municipalities during the initial 2020 wave, temporarily narrowing the desert–adequate gap and diluting baseline associations when pandemic years are incorporated into period averages.

To address the potential temporal mismatch between the 2019 healthcare resource cross-section and the 2015–2019 mortality period, Model C2 was re-estimated, restricting the outcome to 2018–2019 biennium averages. The desert association was virtually identical to the primary estimate (IRR = 1.413; 95% CI: 1.356–1.472; p<0.001; Supplementary Table S6), confirming that the primary findings are robust to potential changes in healthcare infrastructure between 2015 and 2019.

### 3.7. Temporal Trends for 2015–2024

**Figure 3:**
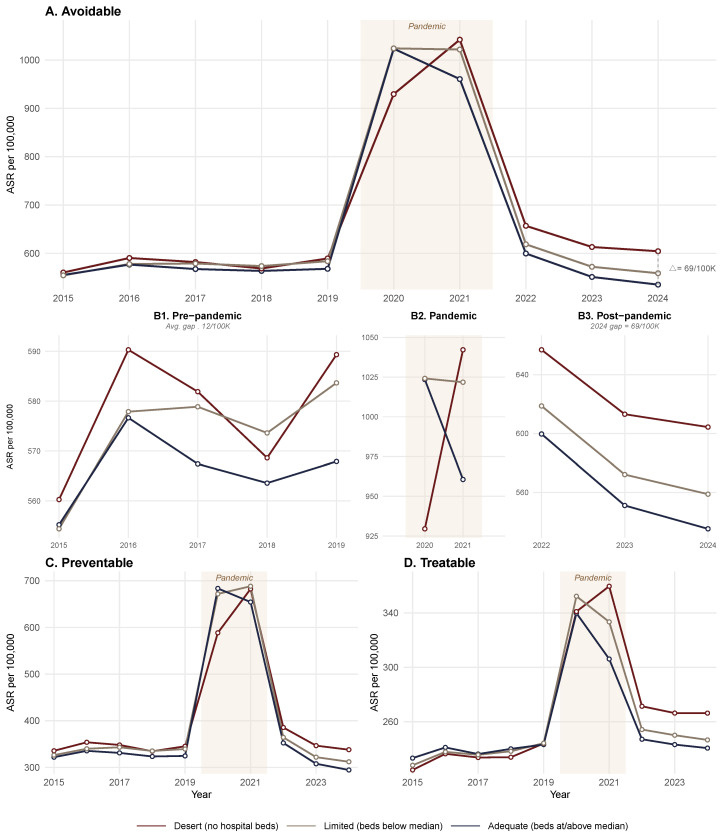
Temporal trends in population-weighted, age-standardized mortality rates by healthcare desert category, Mexico 2015–2024. Panel (**A**): Avoidable mortality (preventable + treatable) across the full study period. Panel (**B1**–**B3**): Zoomed views of avoidable mortality by study phase—pre-pandemic (2015–2019), pandemic (2020–2021), and post-pandemic (2022–2024)—with independent y-axis scales to visualize within-period differentials. Panel (**C**): Preventable mortality. Panel (**D**): Treatable mortality. Rates per 100,000, directly standardized to WHO World Standard Population. The pre-pandemic desert–adequate gap in avoidable mortality (approximately 12 per 100,000 average, 2015–2019) widened to 69 per 100,000 by 2024, representing a fivefold increase. Shaded regions in Panel A indicate pandemic and post-pandemic phases. See Supplementary Table S7 for annual values.

Pre-pandemic avoidable mortality rates were relatively stable during 2015–2019, with desert municipalities exhibiting weighted mean ASR values between 560 and 590 per 100,000 and adequate municipality values between 555 and 577 per 100,000, yielding an average gap of approximately 12 per 100,000 (Figure 3A; Supplementary Table S7).

The pandemic produced a dramatic surge in 2020–2021. During 2020, desert municipalities (930 per 100,000) initially exhibited lower rates than limited (1024) and adequate (1023) municipalities, reflecting the delayed arrival of the first COVID-19 wave in predominantly rural areas. By 2021, the pattern reversed: desert municipalities reached the highest ASR (1042 per 100,000) compared with adequate municipalities (961), a gap of 81 per 100,000.

Post-pandemic rates declined but did not return to pre-pandemic levels. By 2024, desert municipalities recorded an ASR of 604 per 100,000 versus 535 in adequate municipalities—a gap of 69 per 100,000, which is approximately five times wider than the pre-pandemic differential. This widening post-pandemic disparity was observed for both preventable and treatable mortality subcategories (Figure 3C,D), suggesting that the pandemic disproportionately and durably affected municipalities lacking hospital infrastructure.

**Figure 4:**
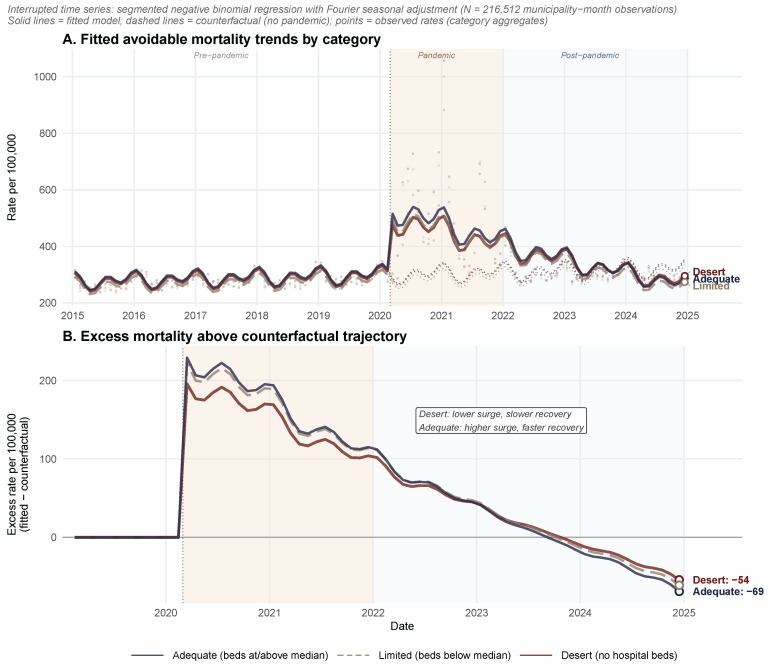
Interrupted time-series analysis of avoidable mortality by healthcare desert category, Mexico, January 2015–December 2024. Panel (**A**): Fitted avoidable mortality trends from segmented negative binomial regression with Fourier seasonal adjustment and cluster-robust standard errors at the municipality level (N=216,512 municipality-month observations). Solid lines represent fitted model trajectories; dotted lines represent counterfactual projections, assuming no pandemic; faded points show observed category-level rates. Panel (**B**): Excess mortality above counterfactual trajectory (fitted minus counterfactual rate per 100,000), highlighting differential pandemic impact and recovery across categories. The pooled interaction model confirmed that the pandemic impact differed significantly by healthcare desert category (likelihood ratio test: $\chi^{2}=133.1$, df = 4, p<0.001). Desert municipalities experienced a lower initial surge (interaction IRR = 0.940; 95% CI: 0.916–0.965; p<0.001), consistent with delayed rural COVID-19 transmission, but exhibited a significantly slower post-pandemic recovery (interaction IRR = 1.002; 95% CI: 1.001–1.003; p<0.001). See Supplementary Table S8 for full model parameters.

The municipal-level interrupted time-series analysis (216,512 municipality-month observations), incorporating Fourier harmonic terms for seasonal adjustment and cluster-robust standard errors at the municipality level, revealed a significant pre-pandemic upward trend in avoidable mortality across all categories (p<0.001; Supplementary Table S8). All four Fourier terms (12-month and 6-month periodicities) were statistically significant (p<0.001), confirming the presence of seasonal variation in mortality. All three categories experienced a significant level change at pandemic onset: adequate municipalities showed an 83.8% surge (IRR = 1.838; 95% CI: 1.798–1.879), limited municipalities surged by 81.8%(IRR = 1.818; 95% CI: 1.774–1.863), and desert municipalities surged by 72.6% (IRR = 1.726; 95% CI: 1.697–1.756). Post-pandemic, all categories showed significant declining slope changes ($\beta_{3}$), but desert municipalities exhibited the smallest monthly decline ($\beta_{3}$ IRR = 0.988; 95% CI: 0.987–0.988) compared with adequate municipalities ($\beta_{3}$ IRR = 0.986; 95% CI: 0.985–0.987). Because the pre-pandemic trend ($\beta_{1}$) was near unity across all categories (IRR ≈ 1.001–1.002), the post-pandemic monthly trend ($\beta_{1}\times \beta_{3}$) closely approximated $\beta_{3}$ alone, confirming that desert municipalities recovered more slowly (Figure 4).

Critically, the pooled interaction model confirmed that the pandemic impact differed significantly across healthcare desert categories (likelihood ratio test: $\chi^{2}=133.1$, df = 4, p<0.001). Desert municipalities experienced a significantly lower initial surge compared with adequate municipalities (interaction IRR = 0.940; 95% CI: 0.916–0.965; p<0.001), consistent with the delayed arrival of COVID-19 in predominantly rural areas. However, desert municipalities exhibited a significantly slower post-pandemic recovery slope (interaction IRR = 1.002; 95% CI: 1.001–1.003; p<0.001), meaning that the monthly rate of mortality decline was attenuated in municipalities lacking hospital infrastructure. Limited municipalities showed no significant difference in initial surge (interaction IRR = 1.013; 95% CI: 0.982–1.045; p=0.418) and a similar recovery slope relative to adequate municipalities (p=0.970). Cumulative excess deaths above counterfactual projections during the pandemic and post-pandemic periods were proportionally highest in limited municipalities (21.0%), followed by adequate (18.1%) and desert municipalities (12.7%), reflecting the initial urban concentration of COVID-19 mortality; however, the slower recovery trajectory in desert municipalities implies that this gap will continue to narrow as the post-pandemic period lengthens.

### 3.8. Spatial Analysis

Global spatial autocorrelation was strongly positive for all mortality indicators: Moran’s I = 0.382 (p<0.001) for avoidable, 0.374 (p<0.001) for preventable, and 0.405 (p<0.001) for treatable mortality. Treatable mortality exhibited the strongest spatial clustering, suggesting that geographic barriers to healthcare access generate particularly concentrated patterns of amenable deaths.

**Figure 5:**
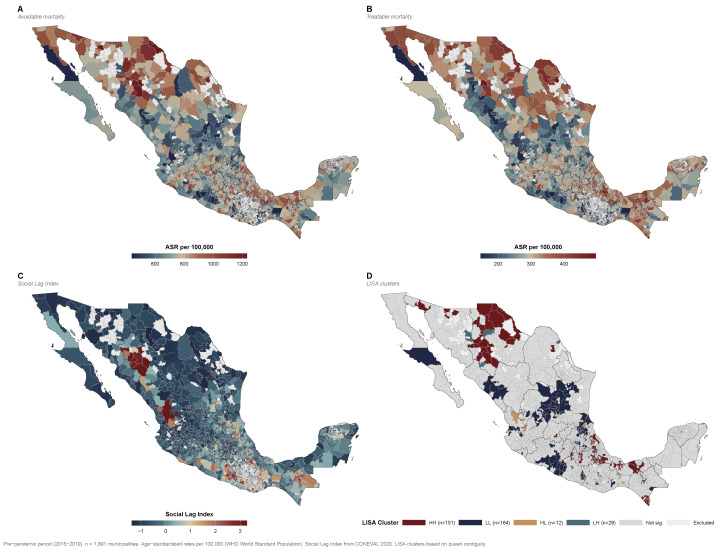
Spatial distribution of avoidable and treatable mortality with local clustering analysis across 1891 Mexican municipalities (pre-pandemic 2015–2019). Panel (**A**): Age-standardized avoidable mortality rate (ASR) per 100,000. Panel (**B**): Age-standardized treatable mortality rate per 100,000. Panel (**C**): Social Lag Index (CONEVAL 2020). Panel (**D**): Local indicators of spatial association (LISA) cluster map for avoidable mortality, identifying 151 High–High hot spots (concentrated in Guerrero, Oaxaca, Chiapas, and the Sierra Madre Occidental), 164 Low–Low cold spots (predominantly in northern metropolitan areas and the Bajío region), 12 High–Low outliers, and 29 Low–High outliers (p<0.05 after 999 conditional permutations with Bonferroni adjustment). Global Moran’s I = 0.382 (p<0.001). Queen contiguity spatial weights matrix.

Local indicators of spatial association (LISA) for avoidable mortality identified 151 High–High municipalities (hot spots), 164 Low–Low municipalities (cold spots), 12 High–Low outliers, and 29 Low–High outliers (Figure 5D). The remaining 1535 municipalities (81.2%) showed no statistically significant local clustering.

High–High clusters were concentrated in southern Mexico—particularly in Guerrero, Oaxaca, and Chiapas—as well as in the Sierra Madre Occidental and portions of the Gulf coast (Figure 5A,D). These regions are characterized by high social deprivation, significant indigenous populations, rugged terrain, and a disproportionate concentration of healthcare deserts. Conversely, Low–Low clusters predominated in northern metropolitan areas and the Bajío region, where greater healthcare investment, lower poverty rates, and better transport infrastructure converge. The spatial concordance between high avoidable mortality clusters (Figure 5D), healthcare desert distribution (Figure 2), and elevated Social Lag Index values (Figure 5C) reinforces the ecological association between infrastructure deficits, social deprivation, and premature avoidable mortality. Treatable mortality (Figure 5B) exhibited a broadly similar spatial pattern to total avoidable mortality (Figure 5A), with the highest rates concentrated in the same southern and mountainous regions.

Moran’s I computed on Pearson residuals of the primary Model C2 was 0.223 (p<0.001), substantially reduced from the raw ASR value of 0.382 but indicating that the multilevel model did not fully capture spatial dependence. A spatial error model (SEM) fitted to log-transformed ASR yielded a spatial autocorrelation parameter of λ=0.507 (p<0.001), confirming substantial spatially structured residual variation. Moran’s I on SEM residuals was −0.045, indicating effective elimination of spatial dependence. In the SEM, hospital beds (β=0.015; p=0.052) and physician density (β=−0.031; p<0.001) retained associations with log(ASR), while the desert category coefficient was negative (β=−0.045; p<0.001; Supplementary Table S9). This directional reversal relative to the primary count model reflects two methodological differences: (1) the SEM outcome (age-standardized rate) already adjusts for age composition, whereas the count model with population offset does not, and (2) the spatial error term absorbs regionally clustered unmeasured confounders that coincide with healthcare desert geography. These complementary results are interpreted in the Discussion.

## 4. Discussion

### 4.1. Summary of Principal Findings

This study provides the first municipal-level ecological analysis of the association between healthcare infrastructure and avoidable mortality across all of Mexico. The principal finding is that healthcare deserts—municipalities lacking any hospital beds, encompassing nearly two-thirds of all analyzed municipalities—were independently associated with 43% higher age-standardized avoidable mortality after adjusting for social deprivation and state-level heterogeneity. This association proved robust across multiple sensitivity analyses: it persisted when restricting the outcome to treatable mortality (IRR = 1.342), when extending the study period to include pandemic and post-pandemic years (IRR = 1.404), when adjusting for age structure (IRR = 1.353), and when restricting to the 2018–2019 biennium to address temporal mismatch in resource data (IRR = 1.413). A spatial error model on log-transformed ASR suggested that the desert association was partly attributable to spatial clustering of risk factors (see Section 4.3). Notably, the interaction between infrastructure deficit and social deprivation was statistically significant (Model D; desert × IRS-z IRR = 0.944, p=0.003), indicating a sub-multiplicative pattern: in healthcare deserts, the marginal effect of additional deprivation is attenuated, consistent with a ceiling effect whereby structural infrastructure absence already dominates the mortality risk profile. Furthermore, the COVID-19 pandemic durably amplified pre-existing disparities: the mortality gap between desert and adequate municipalities widened by approximately fivefold, from 12 per 100,000 pre-pandemic to 69 per 100,000 by 2024.

It is important to note that the healthcare desert classification employed in this study represents a proxy for healthcare infrastructure availability—defined by the presence or absence of hospital beds within municipal boundaries—and does not directly measure functional access to care. Factors such as travel time to the nearest hospital, actual service utilization, care quality, staffing adequacy, equipment availability, and pre-hospital emergency services are not captured by this classification. Consequently, the observed associations reflect the ecological relationship between municipal-level infrastructure availability and population-level mortality patterns rather than a direct measurement of the healthcare access barriers experienced by individuals.

In plain terms, these results indicate that Mexican municipalities without any hospital beds—predominantly small, rural communities concentrated in southern and mountainous regions—experience substantially higher rates of deaths that could have been prevented or treated with timely medical care. This disparity persists even after accounting for differences in poverty and social deprivation between communities. The COVID-19 pandemic further widened this gap: while all communities were affected, those without hospitals recovered more slowly, and by 2024, the mortality difference between communities with and without hospital beds was approximately five times larger than before the pandemic. These findings underscore the need for targeted infrastructure investments in underserved areas, alongside broader efforts to address social determinants of health.

### 4.2. Comparison with Existing Literature

The magnitude of excess mortality observed in Mexican healthcare deserts is consistent with international evidence on the health consequences of infrastructure deficits. Globally, Kruk et al. estimated that 8.6 million annual deaths were amenable to healthcare, with 3.6 million attributable to non-utilization of services [7]; our findings suggest that in Mexico, the non-utilization pathway—driven by the complete absence of hospital infrastructure—constitutes a dominant contributor to avoidable mortality. The GBD Healthcare Access and Quality Index places Mexico in the upper-middle range among nations, yet this aggregate measure masks the extreme within-country heterogeneity documented in the present analysis [45].

Regionally, our results align with ecological studies from other Latin American countries. In Colombia, Rojas-Botero et al. reported significant spatial clustering of COVID-19 mortality associated with multidimensional poverty at the municipal level using comparable LISA methodology [46]. Within Latin American cities, the SALURBAL consortium documented substantial variation in amenable mortality [12,20], though their analysis was limited to urban areas and did not capture the rural–urban infrastructure gradient central to our findings. Within Mexico, Nikoloski et al. analyzed the impact of physician supply on amenable mortality at the state level for 2000–2015, finding protective effects for both general practitioners and specialists [47]; our study extends this work to the municipal level, revealing that the critical infrastructure threshold—the presence or absence of any hospital beds—is the dominant predictor of avoidable mortality beyond aggregate physician counts alone. Similarly, Gómez-Dantés et al. mapped avoidable mortality at the state level [17], and Lozano et al. identified heterogeneity in quality-amenable excess mortality across Mexican states [18]; our analysis demonstrates that within-state variation at the municipal level exceeds what state-level averages suggest, underscoring the need for sub-state resource allocation strategies.

### 4.3. The Physician Paradox

The positive ecological association between physician density and avoidable mortality (IRR = 1.210) reflects a well-documented artifact of ecological studies: physicians—particularly specialists—concentrate in urban referral centers that aggregate complex cases from surrounding areas, simultaneously inflating physician counts and death counts in these municipalities [48,49]. This confounding by indication arises because death certificates record the municipality of usual residence, but the concentration of healthcare resources in referral centers creates an ecological correlation between physician supply and the severity of the case mix treated locally. Our data did not distinguish between primary care physicians and specialists, nor between physicians providing ambulatory versus hospital-based care; Nikoloski et al. previously showed that general practitioner density was protective in Mexico at the state level, while specialist density showed weaker or paradoxical effects [47], consistent with the referral-center mechanism described here. An exploratory urbanicity-adjusted model reduced the physician density IRR from 1.210 to 1.029 (95% CI: 1.009–1.050; Supplementary Table S6), confirming confounding by urban concentration. This model was not designated as the primary specification because log(population) likely mediates the desert→mortality pathway, and controlling for the mediator would inappropriately attenuate the total desert effect.

The spatial error model (SEM) provided complementary evidence on the desert–mortality association. When log-transformed ASR—already age-standardized—was modeled with explicit spatial autocorrelation (λ=0.507), the desert coefficient reversed direction (β=−0.045; p<0.001), physician density showed the expected protective association (β=−0.031; p<0.001), and hospital bed density exhibited a small positive coefficient (β=0.015; p=0.052)—opposite to the protective effect observed in the count model (IRR = 0.921). The reversal of hospital beds reflects the same referral-center confounding: within spatial clusters, municipalities with more beds are urban hubs that aggregate complex cases and recorded deaths from surrounding areas, and once the spatial error term absorbs regional heterogeneity, only this residual urban concentration effect remains. These reversals reflect two factors: (1) the SEM outcome is age-standardized, removing the age-composition confounding present in the count model, and (2) the spatial error term absorbs regionally clustered, unmeasured confounders—including environmental exposures, dietary patterns, and indigenous health determinants—that coincide with healthcare desert geography. Notably, Moran’s I on Model C2 residuals (0.223) confirmed that the primary multilevel model captured only part of the spatial dependence (compared with 0.382 on raw ASR), while the SEM residuals showed effectively no remaining spatial structure (Moran’s I = −0.045). These findings do not contradict the primary results; rather, they indicate that the excess mortality in healthcare deserts reflects a combination of infrastructure deficit and regionally clustered social and environmental determinants, supporting the conclusion that interventions must address both healthcare access and structural socioeconomic factors simultaneously.

### 4.4. Mechanisms and Pathways

Several plausible mechanisms link healthcare desert status to excess avoidable mortality. The most plausible pathway may operate through delayed or forgone access to emergency and hospital-based care for time-sensitive conditions—including acute myocardial infarction, stroke, and complicated childbirth—where hospital proximity is a critical determinant of survival. For treatable conditions such as chronic kidney disease, pneumonia, and diabetes complications, the absence of hospital beds may limit timely inpatient management, potentially contributing to higher mortality from treatable conditions. The stronger protective effect of hospital beds for treatable mortality (IRR = 0.900) compared with all avoidable mortality (IRR = 0.921) supports this interpretation, as does the geographic concordance between treatable mortality hot spots and healthcare desert distribution (Figure 5B,D). Additionally, municipalities without hospitals require patient transfer to neighboring jurisdictions, introducing geographic barriers—distance, cost, and terrain—that disproportionately affect impoverished and indigenous populations [50].

The significant sub-multiplicative interaction between social deprivation and desert status (Model D; desert × IRS-z IRR = 0.944, p=0.003; limited × IRS-z IRR = 0.913, p<0.001) reveals a ceiling effect: in healthcare deserts, the complete absence of hospital infrastructure so thoroughly constrains access to care that additional social deprivation contributes less marginally to excess mortality. In adequate municipalities, where hospitals exist, socioeconomic barriers—including inability to pay, lack of transportation, or limited health literacy—represent the primary determinants of differential mortality; thus, deprivation exerts a stronger marginal effect. This finding suggests that in healthcare deserts, supply-side interventions (hospital construction) may yield larger mortality reductions than demand-side interventions (poverty reduction) alone, whereas in municipalities with existing infrastructure, addressing social determinants may be equally or more effective.

### 4.5. COVID-19 as an Amplifier of Structural Inequalities

The temporal analysis revealed a striking three-phase dynamic. During 2020, desert municipalities initially exhibited lower avoidable mortality than urban areas, consistent with the delayed arrival of the first COVID-19 wave in predominantly rural regions [51]. By 2021, the pattern reversed: desert municipalities recorded the highest mortality rates, likely reflecting the complete absence of hospital surge capacity in these areas. Most critically, the post-pandemic period did not restore the pre-pandemic equilibrium. By 2024, the desert–adequate mortality gap (69 per 100,000) was five times wider than the pre-pandemic differential, consistent with national-level evidence that Mexico’s life expectancy recovery has been incomplete and heterogeneous [52].

The municipal-level interrupted time-series analysis provided quasi-experimental evidence supporting these descriptive trends. All three municipality categories experienced a significant mortality surge at pandemic onset, with level changes of 73–84% above pre-pandemic trajectories after seasonal adjustment (Figure 4). The pooled interaction model (216,512 observations; likelihood ratio of $\chi^{2}=133.1$, p<0.001), incorporating Fourier harmonic terms for seasonality and cluster-robust standard errors, confirmed that the pandemic impact differed significantly by healthcare infrastructure status: desert municipalities experienced a lower initial surge (interaction IRR = 0.940; p<0.001), consistent with delayed rural COVID-19 transmission, but critically exhibited a significantly slower post-pandemic recovery (interaction IRR = 1.002; p<0.001). This differential recovery rate means that while adequate municipalities returned toward pre-pandemic trajectories more rapidly, desert municipalities’ mortality remained elevated for longer—a pattern consistent with the fivefold widening of the descriptively observed mortality gap.

This widening likely reflects both direct mortality and indirect disruptions in chronic disease management, routine vaccinations, and screening programs during and after the pandemic. Indeed, Antonio-Villa et al. demonstrated that non-COVID-19 causes—largely chronic cardiometabolic conditions—accounted for up to one-fifth of excess deaths in Mexico during 2020, with these excess deaths occurring disproportionately out of hospital and clustering in municipalities with higher sociodemographic deprivation [53]. These findings align with the international evidence synthesized by Bambra et al. that the COVID-19 pandemic exacerbated pre-existing health inequalities rather than creating new ones [54].

Notably, Figure 4B shows that by late 2023–2024, excess mortality relative to the counterfactual trajectory turned negative for all categories, indicating that observed death rates fell below what would have been expected had the pandemic not occurred. This pattern is consistent with a partial mortality displacement (harvesting) effect, whereby the pandemic-period deaths disproportionately comprised individuals with advanced chronic conditions whose deaths were accelerated rather than entirely attributable to the pandemic. However, the persistent differential between desert and adequate municipalities—even in this negative-excess phase—suggests that the widened mortality gap cannot be fully explained by harvesting alone. Rather, the structural disruption of healthcare services in municipalities lacking hospital infrastructure may have contributed to sustained impairment of chronic disease management and preventive care that extends beyond the acute pandemic period.

### 4.6. Strengths

This study has several notable strengths. It is the first to examine the healthcare desert–avoidable mortality nexus at the municipal level across all of Mexico, encompassing 1891 municipalities across all 32 federal entities and nearly 5 million death records over 10 years. The use of multilevel modeling with state-level random intercepts accounts for unmeasured contextual heterogeneity. The OECD/Eurostat standardized classification of avoidable deaths enables direct international comparability. Six sensitivity analyses—age-structure adjustment, treatable mortality, full-period regression, temporal mismatch evaluation, spatial error modeling, and physician paradox investigation—provide a comprehensive robustness assessment from multiple analytical angles. The interrupted time-series analysis incorporated Fourier harmonic terms for seasonal adjustment and cluster-robust standard errors to address within-municipality autocorrelation. The spatial error model explicitly addressed the spatial dependence identified by Moran’s I, providing complementary evidence on the mechanisms linking infrastructure deficits to excess mortality. The 10-year span (2015–2024) uniquely captures the complete pre-pandemic, pandemic, and post-pandemic cycle.

### 4.7. Limitations

Several limitations merit consideration. First, as an ecological study, associations observed at the municipal level may not hold at the individual level due to the ecological fallacy [21]. We cannot infer that any individual living in a healthcare desert faces a 43% higher risk of avoidable death; the IRR reflects a population-level association between municipal infrastructure and aggregate mortality. Residents of healthcare deserts may seek care in neighboring municipalities, which would attenuate the true individual-level effect, while deaths occurring in referral hospitals may be registered in the receiving municipality rather than the municipality of residence, potentially biasing associations in either direction. Individual-level risk factors—including tobacco use, alcohol consumption, diet, physical activity, and comorbidity burden—were not available in this ecological design and may confound or modify the observed associations. Similarly, although the CONEVAL “Lack of health access” variable (Table 1) provides a municipal-level proxy for insurance coverage, the substantial changes in affiliation during the study period—particularly following the dissolution of Seguro Popular, when the uninsured share rose from 9% in 2018 to 26% in 2021 [15]—could not be captured dynamically with the available 2020 cross-sectional data. Furthermore, cultural barriers to healthcare utilization—including preference for traditional healers in indigenous communities, language barriers, and distrust of formal institutions—represent unmeasured demand-side factors that may independently reduce healthcare utilization, even when infrastructure is nominally present; conversely, the median poverty rate of 63% in healthcare deserts means that even when private facilities appear in the CLUES registry, they are effectively economically inaccessible to most residents. Because the study design is ecological, we cannot establish that the absence of hospital beds *causes* higher mortality; the observed associations may reflect residual confounding by unmeasured individual or community-level factors despite adjustment for social deprivation. Future studies linking individual death records to healthcare utilization data would help disentangle contextual from compositional effects and establish whether the ecological associations observed here reflect genuine access barriers or residual confounding by unmeasured individual characteristics. Second, healthcare resource data were drawn from a single 2019 cross-section; infrastructure may have changed during the study period, particularly following the INSABI dissolution and the transition to IMSS-Bienestar [55]. However, restricting the analysis to the 2018–2019 biennium—when resource and mortality data are temporally aligned—yielded a virtually identical desert association (IRR = 1.413), mitigating this concern for the pre-pandemic analysis. Third, our healthcare desert classification is based on hospital bed presence as recorded in the CLUES facility directory, which captures the existence of beds but not their functional status, specialty composition (e.g., general vs. intensive care), equipment availability, staffing adequacy, or actual service delivery quality. A municipality classified as “adequate” may harbor beds that are non-operational, understaffed, or lack essential equipment, while conversely, some desert municipalities may have access to nearby emergency transport or ambulance services that partially mitigate the absence of local hospital beds. The DGIS database does not include information on pre-hospital emergency services, ambulance availability, or inter-facility transfer networks, all of which are critical determinants of timely access to hospital care, particularly for time-sensitive conditions. This measurement limitation would bias our estimates toward the null hypothesis(underestimating the true effect of functional infrastructure) because municipalities classified as “adequate” based solely on bed presence may have limited functional capacity. The healthcare desert classification captures the presence or absence of hospital beds within municipal boundaries but does not incorporate geographic distance or travel time to the nearest hospital. Municipalities classified as deserts may vary substantially in their physical proximity to hospital care in neighboring jurisdictions, and this unmeasured heterogeneity likely introduces non-differential misclassification that further biases our estimates toward the null hypothesis. Future studies incorporating geospatial accessibility metrics—such as travel time to the nearest emergency department or two-step floating catchment area analyses—would provide a more refined characterization of healthcare access. Fourth, the Social Lag Index was available only for 2020, introducing a temporal mismatch with the mortality period, though structural deprivation is assumed to be relatively stable over five-year intervals. Fifth, the population threshold (≥1000) excluded 578 very small municipalities, potentially under-representing the most isolated healthcare deserts. Sixth, COVID-19 coding may have displaced deaths from other causes through competing risk mechanisms or misclassification. Seventh, death certificates record the underlying cause of death, which may result in the undercounting of avoidable deaths when contributing causes are not captured, and cause-of-death coding quality may vary across municipalities with different levels of medical certification. Finally, hospital bed presence serves as a proxy for a broader healthcare ecosystem; the absence of beds likely signals concurrent deficiencies in emergency services, surgical capacity, and specialist availability that collectively drive excess mortality.

### 4.8. Policy Implications

These findings carry direct implications for Mexico’s ongoing health-system restructuring under IMSS-Bienestar. The fact that 63% of municipalities lack any hospital beds—and that this deficit is independently associated with 43% excess avoidable mortality—underscores the urgent need for targeted infrastructure expansion. The spatial clustering of high-mortality municipalities in southern Mexico and the Sierra Madre region suggests that regional rather than isolated interventions are needed, i.e., investment in strategically located district hospitals that can serve clusters of underserved municipalities [55]. In the interim, telemedicine platforms, mobile health units, and strengthened primary care networks may partially bridge the access gap for chronic disease management and diagnostic triage. The ceiling effect observed in Model D suggests that in healthcare deserts, where infrastructure absence dominates, hospital construction may yield the largest marginal mortality reductions; in municipalities with existing infrastructure, concurrent social investment and poverty reduction remain critical [50]. Resource allocation formulas should incorporate healthcare desert status alongside existing deprivation indices. The recent collapse in specialized care following the termination of Seguro Popular and INSABI—with hospital discharges for acute myocardial infarction declining by 80.7% and neonatal intensive care by 78.4% among the uninsured between 2018 and 2021 [56]—underscores the fact that the institutional upheaval compounded the structural vulnerability of healthcare deserts during the study period. The pandemic experience further demonstrates that preparedness planning must explicitly address surge capacity in underserved areas, where the post-pandemic mortality gap has proven both durable and wide.

## 5. Conclusions

Healthcare deserts—municipalities completely lacking hospital beds, encompassing 63% of Mexico—are independently associated with 43% higher avoidable mortality (IRR = 1.425; 95% CI: 1.370–1.482), an association that is robust across six sensitivity specifications (IRR range: 1.342–1.413). Hospital bed availability showed a protective dose–response relationship (7.9% lower mortality per SD increase). The significant sub-multiplicative interaction between social deprivation and desert status indicates a ceiling effect: in healthcare deserts, structural infrastructure absence dominates the mortality risk profile, attenuating the marginal impact of additional deprivation, suggesting that hospital construction may yield the largest mortality gains in these municipalities. The COVID-19 pandemic durably amplified pre-existing disparities: by 2024, the desert–adequate mortality gap was approximately five times wider than the pre-pandemic differential, with strong spatial clustering concentrated in southern Mexico and mountainous regions. These findings provide an evidence base for the restructuring of Mexico’s health system under IMSS-Bienestar, supporting targeted regional hospital investments guided by spatial analysis—rather than uniform national policies—alongside concurrent action on social determinants of health.

## Figures and Tables

**Table 1:** Descriptive characteristics of 1891 Mexican municipalities by healthcare desert category (pre-pandemic period, 2015–2019 averages).

Variable	Overall (*n* = 1891)	Desert (*n* = 1187)	Limited (*n* = 306)	Adequate (*n* = 398)	*p*-Value ^†^
Demographics					
Municipalities, *n* (%)	1891 (100)	1187 (62.8)	306 (16.2)	398 (21.0)	—
Population	12,775 [4617–37,947]	6881 [2780–14,642]	55,111 [31,661–117,606]	29,453 [12,466–100,471]	<0.001
Mortality rates (per 100,000)					
ASR avoidable mortality	754.0 [658.5–852.6]	748.6 [648.6–854.2]	770.8 [687.7–856.2]	753.9 [664.6–846.3]	0.075
ASR preventable mortality	457.1 [393.5–519.7]	453.8 [388.1–523.4]	465.6 [408.2–518.0]	455.5 [395.2–509.6]	0.155
ASR treatable mortality	297.0 [255.4–340.6]	293.6 [250.5–340.6]	303.1 [273.7–339.2]	301.4 [260.9–341.1]	0.017
Average annual deaths < 75 y	69 [33–161]	44 [25–78]	222 [138–398]	136 [68–385]	<0.001
Healthcare resources					
Health facilities	7 [4–14]	5 [3–8]	17 [11–26]	13 [8–23]	<0.001
Hospitals	0 [0–1]	0 [0–0]	1 [1–1]	1 [1–3]	<0.001
Hospital beds per 1000	0.0 [0.0–0.7]	0.0 [0.0–0.0]	0.4 [0.2–0.6]	1.2 [0.9–1.9]	<0.001
Physicians per 1000	1.3 [0.8–2.1]	1.0 [0.6–1.7]	1.2 [0.9–1.6]	2.3 [1.7–3.3]	<0.001
Nurses per 1000	2.0 [1.2–3.5]	1.6 [1.0–2.6]	2.0 [1.5–2.7]	4.1 [3.1–5.8]	<0.001
Social deprivation					
Social Lag Index ^‡^	−0.11 (1.00)	0.06 (1.02)	−0.31 (0.89)	−0.43 (0.90)	<0.001
Poverty rate (%)	59.7 [44.0–76.4]	63.2 [48.2–80.4]	55.0 [42.1–68.9]	50.0 [35.3–66.0]	<0.001
Extreme poverty rate (%)	10.9 [5.0–21.4]	12.8 [5.9–24.7]	10.1 [4.7–19.0]	7.7 [3.2–15.7]	<0.001
Lack of health access (%)	24.8 [17.4–34.1]	24.3 [16.5–34.8]	27.7 [21.0–36.1]	24.5 [18.6–31.4]	<0.001

Values are medians [Q1–Q3] unless otherwise noted. ^†^ Kruskal–Wallis test comparing distributions across the three healthcare desert categories. ^‡^ Mean (SD). Healthcare desert classification: Desert = no hospital beds; Limited = hospital beds below median (0.69 per 1000); Adequate = hospital beds at or above median (reference). ASR: age-standardized rate per 100,000 population; direct method using WHO World Standard Population. Social Lag Index from CONEVAL 2020. Lack of health access (%): percentage of the municipal population without affiliation with any health service (IMSS, ISSSTE, Seguro Popular/INSABI, or private insurance), per CONEVAL 2020. Poverty data from CONEVAL 2020 (n = 1890 due to one missing value).

**Table 4:** Sensitivity analyses: negative binomial regression models comparing primary pre-pandemic analysis with treatable mortality and full-period specifications (Model C2, state-level random intercept).

Predictor	Pre-Pandemic Avoidable (2015–2019)	Pre-Pandemic Treatable (2015–2019)	Full-Period Avoidable (2015–2024)
Social Lag Index (per SD)	1.025 (1.007–1.043) p=0.006	1.001 (0.986–1.015) p=0.942	1.000 (0.983–1.016) p=0.960
Physicians per 1000 (per SD)	1.210 (1.178–1.243) p<0.001	1.198 (1.161–1.237) p<0.001	1.192 (1.163–1.222) p<0.001
Hospital beds per 1000 (per SD)	0.921 (0.895–0.949) p<0.001	0.900 (0.871–0.931) p<0.001	0.933 (0.909–0.958) p<0.001
Limited (vs. Adequate)	0.948 (0.907–0.992) p=0.021	0.907 (0.871–0.944) p<0.001	0.959 (0.919–1.001) p=0.054
Desert (vs. Adequate)	1.425 (1.370–1.482) p<0.001	1.342 (1.295–1.391) p<0.001	1.404 (1.353–1.457) p<0.001
AIC	16,817	—	17,153

Values are IRRs (95% CIs). Pre-pandemic avoidable: primary model (Model C2) from Table 3, shown for comparison. Pre-pandemic treatable: outcome restricted to treatable (healthcare-amenable) causes of death. Full-period avoidable: model re-estimated using 2015–2024 annual averages (n=1891 municipalities across all 32 federal entities). All models include state-level random intercept and log(population) offset. Continuous predictors standardized as z-scores.

## Data Availability

All data used in this study are publicly available from official Mexican government sources: mortality microdata from INEGI (https://www.datos.gob.mx/, accessed on 25 February 2025), healthcare resource data from DGIS, Social Lag Index from CONEVAL, and population projections from CONAPO. Analysis scripts are available at https://doi.org/10.5281/zenodo.19241171.

## Supplementary Materials

The following supporting information can be downloaded at: https://www.mdpi.com/article/10.3390/healthcare14070890/s1, Figure S1: Weekly stacked bar chart of avoidable deaths by cause group; Table S1: Cause-specific avoidable mortality by pandemic phase; Table S2: Extended descriptive characteristics by desert category; Table S3: Spearman rank correlation matrix; Table S4: Model fit comparison (AIC and BIC); Table S5: Age-structure adjusted regression model (C2a); Table S6: Additional sensitivity analyses; Table S7: Population-weighted annual mortality rates 2015–2024; Table S8: Interrupted time series analysis; Table S9: OLS and spatial error model comparison.

## Abbreviations

The following abbreviations are used in this manuscript:

AIC	Akaike’s Information Criterion
ASR	Age-standardized rate
BIC	Bayesian Information Criterion
CLUES	Unique Health Establishment Code (*Clave Única de Establecimientos de Salud*)
CONAPO	National Population Council (*Consejo Nacional de Población*)
CONEVAL	National Council for the Evaluation of Social Development Policy
DGIS	General Directorate of Health Information (*Dirección General de Información en Salud*)
EDR	Registered Deaths Statistics (*Estadísticas de Defunciones Registradas*)
ICD-10	International Classification of Diseases, Tenth Revision
ICC	Intraclass correlation coefficient
IMSS	Mexican Social Security Institute
INEGI	National Institute of Statistics and Geography
IRR	Incidence rate ratio
IRS	Social Lag Index (*Índice de Rezago Social*)
ISSSTE	Institute of Security and Social Services for State Workers
ITS	Interrupted time series
LISA	Local indicators of spatial association
LMIC	Low- and middle-income country
NB	Negative binomial
OECD	Organisation for Economic Co-operation and Development
SAR	Spatial autoregressive model
SEM	Spatial error model
SSa	Secretariat of Health (*Secretaría de Salud*)
VIF	Variance inflation factor
WHO	World Health Organization
