# Supplementary material for: Healthcare Deserts and Avoidable Mortality in Mexico: A Municipal-Level Ecological Analysis of Health System Resources, Social Deprivation, and Preventable Deaths, 2015–2024
Source: Healthcare (Basel). 2026 Mar 31;14(7):890. doi: 10.3390/healthcare14070890 (PMC13073501; doi:10.3390/healthcare14070890)
Supplement: Supplementary file 1 [file healthcare-14-00890-s001.zip › healthcare-4192895-supplementary.pdf]

Supplementary Material

Contents

Supplementary Figure S1	Weekly stacked bar chart of avoidable deaths by cause group
Supplementary Table S1	Cause-specific avoidable mortality by pandemic phase
Supplementary Table S2	Extended descriptive characteristics by desert category
Supplementary Table S3	Spearman rank correlation matrix
Supplementary Table S4	Model fit comparison (AIC and BIC)
Supplementary Table S5	Age-structure adjusted regression model (C2a)
Supplementary Table S6	Additional sensitivity analyses
Supplementary Table S7	Population-weighted annual mortality rates 2015–2024
Supplementary Table S8	Interrupted time series analysis
Supplementary Table S9	OLS and spatial error model comparison

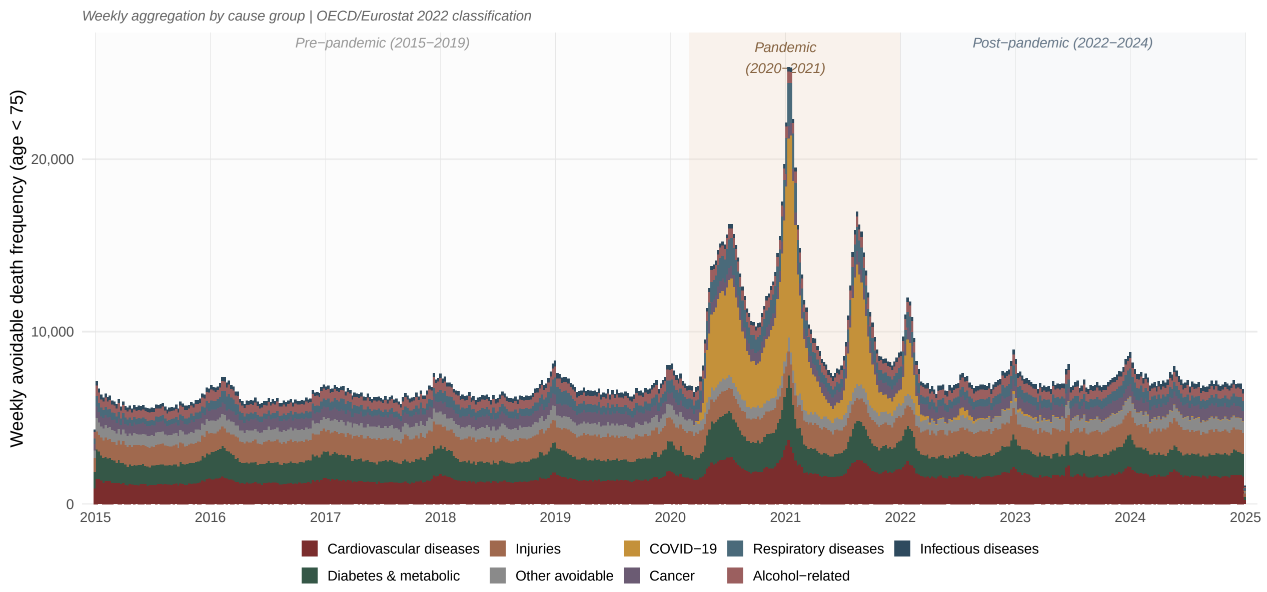

Supplementary Figure S1: Weekly stacked bar chart of avoidable deaths by cause group across 523 ISO weeks, Mexico 2015–2024. Each bar represents one ISO week; colour segments correspond to the nine major avoidable cause groups. A total of 1,485 deaths (<0.04%) were excluded due to unknown day of occurrence.

Supplementary Table S1: Cause-specific avoidable mortality by pandemic phase: total deaths, annual averages, preventable/treatable composition, and percentage change relative to the pre-pandemic period, Mexico 2015–2024 (deaths < 75 years, $N = 4{,}028{,}038$ avoidable deaths).

Cause group	Phase	Total deaths	Annual avg.	Preventable	Treatable	% avoidable†	% change‡
**Cardiovascular diseases**							
	Pre-pandemic	346,419	69,284	168,509	177,910	20.7	—
	Pandemic	213,467	106,734	104,900	108,568	17.6	+54.1
	Post-pandemic	266,589	88,863	130,105	136,484	23.3	+28.3
**Diabetes & metabolic**							
	Pre-pandemic	338,747	67,749	168,318	170,428	20.3	—
	Pandemic	193,700	96,850	96,396	97,304	16.0	+43.0
	Post-pandemic	212,915	70,972	105,677	107,238	18.6	+4.8
**Injuries**							
	Pre-pandemic	325,477	65,095	325,477	0	19.5	—
	Pandemic	140,011	70,006	140,011	0	11.6	+7.5
	Post-pandemic	211,819	70,606	211,819	0	18.5	+8.5
**COVID-19**§							
	Pandemic	334,720	167,360	334,720	0	27.6	—
	Post-pandemic	24,989	8,330	24,989	0	2.2	—
**Cancer**							
	Pre-pandemic	170,560	34,112	86,160	84,400	10.2	—
	Pandemic	73,535	36,768	35,658	37,877	6.1	+7.8
	Post-pandemic	112,158	37,386	52,719	59,439	9.8	+9.6
**Respiratory diseases**							
	Pre-pandemic	110,000	22,000	39,575	70,425	6.6	—
	Pandemic	97,139	48,570	15,406	81,733	8.0	+120.8
	Post-pandemic	79,951	26,650	19,010	60,941	7.0	+21.1
**Alcohol-related**							
	Pre-pandemic	135,094	27,019	135,094	0	8.1	—
	Pandemic	58,962	29,481	58,962	0	4.9	+9.1
	Post-pandemic	81,126	27,042	81,126	0	7.1	+0.1
**Infectious diseases**							
	Pre-pandemic	63,677	12,735	44,224	19,452	3.8	—
	Pandemic	26,806	13,403	16,518	10,288	2.2	+5.2
	Post-pandemic	45,981	15,327	26,169	19,812	4.0	+20.3
**Maternal & perinatal**							
	Pre-pandemic	66,361	13,272	2	66,359	4.0	—
	Pandemic	22,681	11,341	0	22,681	1.9	−14.6
	Post-pandemic	31,738	10,579	1	31,737	2.8	−20.3
**Genitourinary diseases**							
	Pre-pandemic	47,724	9,545	0	47,724	2.9	—
	Pandemic	21,271	10,636	0	21,271	1.8	+11.4
	Post-pandemic	35,492	11,831	0	35,492	3.1	+23.9
**Digestive diseases**							
	Pre-pandemic	28,020	5,604	0	28,020	1.7	—
	Pandemic	13,424	6,712	0	13,424	1.1	+19.8
	Post-pandemic	21,208	7,069	0	21,208	1.8	+26.1
**Congenital malformations**							
	Pre-pandemic	25,527	5,105	1,904	23,623	1.5	—
	Pandemic	8,797	4,399	633	8,164	0.7	−13.8
	Post-pandemic	12,192	4,064	780	11,412	1.1	−20.4
**Nervous system diseases**							
	Pre-pandemic	9,156	1,831	0	9,156	0.5	—
	Pandemic	4,447	2,224	0	4,447	0.4	+21.4
	Post-pandemic	6,944	2,315	0	6,944	0.6	+26.4
**Drug-related**							
	Pre-pandemic	2,244	449	2,244	0	0.1	—
	Pandemic	1,400	700	1,400	0	0.1	+56.0
	Post-pandemic	2,179	726	2,179	0	0.2	+61.8
**Adverse medical effects**							
	Pre-pandemic	1,493	299	0	1,493	0.1	—
	Pandemic	647	324	0	647	0.1	+8.3
	Post-pandemic	1,251	417	0	1,251	0.1	+39.7

Cause groups ranked by total deaths across all phases (excluding COVID-19). Avoidable mortality classified per OECD/Eurostat 2022 framework: preventable deaths are amenable to public health interventions; treatable deaths are amenable to healthcare system interventions. The OECD/Eurostat 50/50 split rule applies at the individual ICD-10 code level for dual-classified causes (e.g., ischaemic heart disease); when aggregated by cause group, preventable and treatable subtotals may differ because each group also contains codes classified as exclusively preventable or exclusively treatable. † Percentage of total avoidable deaths within each phase. ‡ Percentage change in annual average relative to pre-pandemic (2015–2019); positive values indicate increase. § COVID-19 coded under ICD-10 U07.1–U07.2 (provisional assignment of new diseases); classified as 100% preventable per OECD/Eurostat framework. No pre-pandemic baseline exists for COVID-19. Phase classification: Pre-pandemic (2015–2019, 5 years), Pandemic (2020–2021, 2 years), Post-pandemic (2022–2024, 3 years).

Supplementary Table S2: Extended descriptive characteristics of 1,891 municipalities by healthcare desert category: complete variable set with summary statistics (pre-pandemic 2015–2019).

Variable	Overall (N = 1,891)	Desert (n = 1,187)	Limited (n = 306)	Adequate (n = 398)
**Demographics**				
Population	12,775 [4,617–37,947]	6,881 [2,780–14,642]	55,111 [31,661–117,606]	29,453 [12,466–100,471]
**Mortality outcomes**				
ASR avoidable (per 100k)	754.0 [658.5–852.6]	748.6 [648.6–854.2]	770.8 [687.7–856.2]	753.9 [664.6–846.3]
ASR preventable (per 100k)	457.1 [393.5–519.7]	453.8 [388.1–523.4]	465.6 [408.2–518.0]	455.5 [395.2–509.6]
ASR treatable (per 100k)	297.0 [255.4–340.6]	293.6 [250.5–340.6]	303.1 [273.7–339.2]	301.4 [260.9–341.1]
Avg. annual deaths <75 y	69.0 [33.4–160.7]	43.8 [25.2–78.0]	221.7 [138.1–398.3]	136.1 [68.2–385.0]
Avg. annual avoidable deaths	55.2 [26.5–128.5]	34.6 [20.2–63.2]	177.5 [109.6–328.2]	111.0 [55.0–310.4]
**Healthcare resources (absolute)**				
Health facilities, n	7 [4–14]	5 [3–8]	17 [11–26]	13 [8–23]
Hospitals, n	0 [0–1]	0 [0–0]	1 [1–1]	1 [1–3]
Hospital beds, n	0 [0–17]	0 [0–0]	18 [12–36]	40 [18–133]
Total physicians, n	15 [6–46]	8 [4–14]	65 [42–119]	65 [30–226]
Total nurses, n	23 [9–80]	11 [6–20]	111 [72–199]	125 [55–485]
Outpatient clinics, n	13 [6–29]	8 [5–13]	38 [26–65]	35 [19–87]
**Healthcare resources (per 1,000 pop.)**				
Hospital beds per 1,000	0.0 [0.0–0.7]	0.0 [0.0–0.0]	0.4 [0.2–0.6]	1.2 [0.9–1.9]
Physicians per 1,000	1.3 [0.8–2.1]	1.0 [0.6–1.7]	1.2 [0.9–1.6]	2.3 [1.7–3.3]
Nurses per 1,000	2.0 [1.2–3.5]	1.6 [1.0–2.6]	2.0 [1.5–2.7]	4.1 [3.1–5.8]
**Social deprivation**				
Social Lag Index†	−0.11 (1.00)	0.06 (1.02)	−0.31 (0.89)	−0.43 (0.90)
Poverty rate (%)	59.7 [44.0–76.4]	63.2 [48.2–80.4]	55.0 [42.1–68.9]	50.0 [35.3–66.0]
Extreme poverty rate (%)	10.9 [5.0–21.4]	12.8 [5.9–24.7]	10.1 [4.7–19.0]	7.7 [3.2–15.7]
Lack of health access (%)	24.8 [17.4–34.1]	24.3 [16.5–34.8]	27.7 [21.0–36.1]	24.5 [18.6–31.4]

Values are median [Q1–Q3] unless otherwise noted. † Mean (SD). Healthcare desert classification: Desert = no hospital beds; Limited = hospital beds below median (0.69 per 1,000); Adequate = hospital beds at or above median. ASR: age-standardized rate per 100,000 population, direct method using WHO World Standard Population. All Kruskal–Wallis tests comparing distributions across categories yielded $p < 0.001$, except ASR avoidable ($p = 0.075$), ASR preventable ($p = 0.155$), and ASR treatable ($p = 0.017$). Social Lag Index and poverty data from CONEVAL 2020. Healthcare resources from DGIS Sectorial Resources 2019.

Supplementary Table S3: Spearman rank correlation matrix among continuous predictors and age-standardized mortality rates across 1,891 municipalities (pre-pandemic 2015–2019).

	ASR avoid.	ASR prev.	ASR treat.	Beds/1k	Docs/1k	Soc. Lag	Poverty	Population
ASR avoidable	1.000							
ASR preventable	0.931	1.000						
ASR treatable	0.833	0.596	1.000					
Beds/1,000	0.003	0.003	0.004	1.000				
Docs/1,000	−0.179	−0.133	−0.201	0.442	1.000			
Social Lag Index	0.065	0.172	−0.127	−0.135	−0.062	1.000		
Poverty (%)	0.111	0.157	0.009	−0.156	−0.150	0.844	1.000	
Population	0.110	0.037	0.204	0.407	−0.171	−0.381	−0.313	1.000

Lower triangular matrix of Spearman rank correlation coefficients (ρ). ASR: age-standardized rate per 100,000 population. Beds/1,000: hospital beds per 1,000 population. Docs/1,000: physicians per 1,000 population. Social Lag Index from CONEVAL 2020. Correlations with $|\rho| > 0.05$ are statistically significant at $p < 0.05$ given $n = 1{,}891$.

Supplementary Table S4: Model fit comparison (AIC and BIC) for negative binomial regression specifications in the pre-pandemic (2015–2019) and full-period (2015–2024) analyses.

Model	Period	AIC	BIC
A — Social deprivation only	2015–2019	17,781.3	17,797.9
B — Healthcare resources only	2015–2019	17,093.2	17,126.5
C — Full (fixed effects)	2015–2019	17,074.7	17,113.5
**C2 — Full (mixed, state RE)**	**2015–2019**	**16,817.1**	**16,861.5**
D — Full + interaction	2015–2019	17,066.5	17,116.4
C — Full (fixed effects)	2015–2024	17,431.1	17,470.0
**C2 — Full (mixed, state RE)**	**2015–2024**	**17,152.5**	**17,196.9**

Bold rows indicate the best-fitting model within each period (lowest AIC). Model specifications — A: Social Lag Index (z-score) only; B: physician density (z-score), hospital bed density (z-score), and healthcare desert category; C: full model combining A and B covariates; C2: full model with state-level random intercept; D: full model with social deprivation × desert category interaction terms. RE: random effect. All models use log(average annual population) as offset and negative binomial distribution. Models A–D estimated for the pre-pandemic period only; C and C2 re-estimated for the full 2015–2024 period as sensitivity analysis.

Supplementary Table S5: Age-structure adjusted negative binomial regression model (Model C2a) for avoidable mortality: incidence rate ratios with 95% confidence intervals (pre-pandemic 2015–2019, n = 1,891 municipalities).

Predictor	Model C2 (primary)	Model C2a (age-adjusted)
Social Lag Index (per SD)	1.025 (1.007–1.043); $p = 0.006$	1.069 (1.054–1.085); $p < 0.001$
Physicians per 1,000 (per SD)	1.210 (1.178–1.243); $p < 0.001$	1.146 (1.119–1.173); $p < 0.001$
Hospital beds per 1,000 (per SD)	0.921 (0.895–0.949); $p < 0.001$	0.932 (0.910–0.955); $p < 0.001$
Limited (vs. Adequate)	0.948 (0.907–0.992); $p = 0.021$	0.979 (0.945–1.015); $p = 0.251$
Desert (vs. Adequate)	1.425 (1.370–1.482); $p < 0.001$	1.353 (1.312–1.396); $p < 0.001$
Prop. elderly 60–74 y (per SD)	—	1.220 (1.204–1.237); $p < 0.001$
ΔAIC vs Model C2	(reference)	−664

Model C2 is the primary model from Table 3, shown for comparison. Model C2a extends Model C2 by incorporating the standardized proportion of the under-75 population aged 60–74 years (prop-elderly-z) as a covariate to account for differential age composition across municipalities. This adjustment addresses the methodological concern that the negative binomial count model with population offset adjusts for population volume but not age structure, whereas descriptive statistics report age-standardized rates (ASR). Both models include state-level random intercepts and log(average annual population) offset. IRR = $\exp(\beta)$; continuous predictors standardized as z-scores. * ΔAIC to be verified from R output; the substantial improvement indicates that age composition explains meaningful residual variance in avoidable mortality.

Supplementary Table S6: Additional sensitivity analyses: negative binomial regression models for avoidable mortality under alternative specifications (all with state-level random intercept or state fixed effects).

Predictor	Primary (2015–2019)	2018–2019 only	Urbanicity-adjusted†
Social Lag Index (per SD)	1.025 (1.007–1.043)	1.020 (1.002–1.039)	0.986 (0.975–0.998)
Physicians per 1,000 (per SD)	1.210 (1.178–1.243)	1.199 (1.166–1.233)	1.029 (1.009–1.050)
Hospital beds per 1,000 (per SD)	0.921 (0.895–0.949)	0.924 (0.897–0.952)	0.979 (0.960–0.999)
Limited (vs. Adequate)	0.948 (0.907–0.992)	0.949 (0.905–0.995)	0.978 (0.953–1.003)
Desert (vs. Adequate)	1.425 (1.370–1.482)	1.413 (1.356–1.472)	1.009 (0.982–1.037)
Prop. elderly 60–74 y (per SD)	—	—	1.136 (1.123–1.148)
log(population) (per SD)	—	—	0.763 (0.753–0.773)

Values are IRR (95% CI). Primary model (Model C2 from Table 3) shown for comparison. 2018–2019 only: restricts the outcome to the 2018–2019 biennium to address temporal alignment between 2019 resource data and mortality data; fitted using `glm.nb` with state fixed effects due to mixed-model convergence constraints. The full-period sensitivity analysis (2015–2024) is reported in Table 4. † Urbanicity-adjusted: exploratory model incorporating log-transformed population as a proxy for urban concentration and prop-elderly-z to empirically investigate the physician density paradox; this specification is not designated as primary because log(population) likely mediates the desert→mortality pathway (rural municipalities are simultaneously smaller, more likely to lack hospitals, and have fewer physicians). All models use log(population) offset and negative binomial distribution. Continuous predictors standardized as z-scores.

Supplementary Table S7: Population-weighted annual age-standardized mortality rates per 100,000 by healthcare desert category and mortality subcategory, Mexico 2015–2024.

		Avoidable			Treatable		
Year	Phase	Desert	Limited	Adequate	Desert	Limited	Adequate
2015	Pre-pandemic	560.3	554.4	555.2	224.6	228.0	233.3
2016	Pre-pandemic	590.3	577.9	576.7	236.4	237.9	241.1
2017	Pre-pandemic	581.9	578.9	567.4	233.7	235.7	236.3
2018	Pre-pandemic	568.6	573.6	563.6	234.0	238.3	240.1
2019	Pre-pandemic	589.3	583.7	567.9	244.0	244.3	243.3
2020	Pandemic	929.5	1,024.2	1,023.5	341.0	352.3	339.8
2021	Pandemic	1,042.2	1,021.8	960.6	359.6	333.4	306.1
2022	Post-pandemic	656.9	618.7	599.6	271.4	254.3	247.1
2023	Post-pandemic	613.1	572.1	551.1	266.4	250.1	243.2
2024	Post-pandemic	604.3	558.8	535.2	266.4	246.7	240.6

Rates are population-weighted mean age-standardized rates per 100,000, computed using direct standardization with WHO World Standard Population weights. Healthcare desert categories based on 2019 DGIS hospital bed availability: Desert = no hospital beds; Limited = below median; Adequate = at or above median. Phase classification: Pre-pandemic (2015–2019), Pandemic (2020–2021), Post-pandemic (2022–2024). Number of municipalities per category: Desert n = 1,181–1,187; Limited n = 306; Adequate n = 398. Avoidable mortality = preventable + treatable per OECD/Eurostat 2022 classification.

Supplementary Table S8: Interrupted time series analysis of avoidable mortality by healthcare desert category: segmented negative binomial regression with Fourier seasonal adjustment and cluster-robust standard errors at the municipality level, Mexico January 2015–December 2024.

Panel A. Stratified models (separate model per healthcare desert category)

Parameter	Description	Adequate (n = 47,137)	Limited (n = 36,693)	Desert (n = 132,682)
β_1	Pre-pandemic trend (per month)	1.002 (1.001–1.002) $p < 0.001$	1.002 (1.002–1.003) $p < 0.001$	1.001 (1.001–1.002) $p < 0.001$
β_2	Level change at pandemic onset	1.838 (1.798–1.879) $p < 0.001$	1.818 (1.774–1.863) $p < 0.001$	1.726 (1.697–1.756) $p < 0.001$
β_3	Post-pandemic slope change (per month)	0.986 (0.985–0.987) $p < 0.001$	0.985 (0.984–0.985) $p < 0.001$	0.988 (0.987–0.988) $p < 0.001$
	Model AIC	242,290	208,119	520,509

Panel B. Interaction model (pooled, n = 216,512 municipality-month observations)

Parameter	Description	IRR (95% CI)	p-value
**Main effects (Adequate = reference)**			
β_1	Pre-pandemic trend (per month)	1.001 (1.001–1.002)	<0.001
β_2	Level change at pandemic onset	1.826 (1.788–1.865)	<0.001
β_3	Post-pandemic slope change (per month)	0.986 (0.985–0.987)	<0.001
	Limited (baseline difference)	0.940 (0.912–0.969)	<0.001
	Desert (baseline difference)	0.982 (0.956–1.009)	0.187
**Interaction terms**			
$\beta_2\times$ Limited	Differential level change (Limited vs Adequate)	1.013 (0.982–1.045)	0.418
$\beta_2\times$ Desert	Differential level change (Desert vs Adequate)	0.940 (0.916–0.965)	<0.001
$\beta_3\times$ Limited	Differential slope change (Limited vs Adequate)	1.000 (0.999–1.001)	0.970
$\beta_3\times$ Desert	Differential slope change (Desert vs Adequate)	1.002 (1.001–1.003)	<0.001
	Likelihood ratio test vs model without interactions	$\chi^2 = 133.1$, df = 4, $p < 0.001$	

Segmented negative binomial regression with monthly avoidable death counts as outcome and log(monthly population) as offset. The intervention was defined at March 2020 (time index = 63). Fourier harmonic terms at 12-month and 6-month periodicities (sin and cos pairs) were included to control for seasonal variation in mortality; all four Fourier terms were statistically significant at $p < 0.001$ across all models. Standard errors were computed using cluster-robust sandwich estimators at the municipality level to account for within-municipality temporal autocorrelation. β_1 captures the pre-pandemic monthly trend, β_2 estimates the immediate level change at pandemic onset, and β_3 estimates the change in monthly trend post-intervention. Interaction terms test whether the pandemic impact differed by desert category (Adequate = reference). IRR: incidence rate ratio; CI: confidence interval. AIC: Akaike Information Criterion.

Supplementary Table S9: Comparison of ordinary least squares (OLS) and spatial error model (SEM) regression on log-transformed age-standardized avoidable mortality rate (n = 1,891 municipalities, pre-pandemic 2015–2019).

Predictor	OLS β (SE)	p-value	SEM β (SE)	p-value
(Intercept)	6.642 (0.013)	<0.001	6.652 (0.014)	<0.001
Social Lag Index (per SD)	0.013 (0.006)	0.015	0.014 (0.007)	0.053
Physicians per 1,000 (per SD)	−0.049 (0.009)	<0.001	−0.031 (0.008)	<0.001
Hospital beds per 1,000 (per SD)	0.025 (0.009)	0.004	0.015 (0.008)	0.052
Limited (vs. Adequate)	−0.001 (0.018)	0.949	−0.004 (0.016)	0.794
Desert (vs. Adequate)	−0.034 (0.015)	0.020	−0.045 (0.013)	<0.001
**Spatial diagnostics**				
λ (spatial autocorrelation)	—	—	0.507	<0.001
Moran's I on residuals	—‡	—	NS‡	

OLS: ordinary least squares linear regression. SEM: spatial error model estimated by maximum likelihood via the `spatialreg` R package. Outcome: log-transformed age-standardized avoidable mortality rate (per 100,000). Queen contiguity spatial weights matrix with row-standardization. λ = spatial error autocorrelation parameter governing spatially structured unobserved factors. Coefficients represent the change in log(ASR) per unit change in each predictor. Continuous predictors standardized as z-scores. Desert and Limited are indicator variables relative to Adequate (reference). ‡ For context: Moran's I on raw ASR was 0.382 ($p < 0.001$). The non-significant Moran's I on SEM residuals confirms effective removal of spatial dependence. The directional reversal of the desert coefficient relative to the primary count model (Table 3, IRR = 1.425) reflects two methodological differences: (1) the SEM uses age-standardized rates (already adjusted for age composition) rather than raw counts with population offset; and (2) the spatial error term ($\lambda = 0.507$) absorbs regionally clustered unmeasured confounders that are spatially coincident with healthcare desert geography. NS: not significant.
